# Will the Inducing and Maintaining Remission of Non-biological Agents and Biological Agents Differ for Crohn's Disease? The Evidence From the Network Meta-Analysis

**DOI:** 10.3389/fmed.2021.679258

**Published:** 2021-09-01

**Authors:** Mingjun Rui, Zhengyang Fei, Yingcheng Wang, Fenghao Shi, Rui Meng, Ye Shang, Aixia Ma, Hongchao Li

**Affiliations:** ^1^School of International Pharmaceutical Business, China Pharmaceutical University, Nanjing, China; ^2^Center for Pharmacoeconomics and Outcomes Research, China Pharmaceutical University, Nanjing, China

**Keywords:** Crohn's disease, network meta-analysis, anti-inflammatory agents, immunosuppressive agents, anti-tumor necrosis factor, biologic agents, steroids

## Abstract

**Background:** Several drugs currently are available for the treatment of Crohn's disease, including non-biological agents such as anti-inflammatory agents, steroids, immunosuppressive agents, and biologic agents such as anti-tumor necrosis factor (TNF), anti-α4β7 integrin, anti-alpha-4 integrin and anti-interleukin 12/23. However, the choice of treatments for induction and maintenance is still a challenge. The relevant comparison between non-biologic agents and biologic agents is few. In our research, we aimed to help making decisions, as well as providing clinicians and patients with medication references.

**Methods:** We searched MEDLINE, Embase, and the Cochrane Central Register of controlled trials for relevant randomized controlled trials published through to July 2020 and systematic reviews published from January 2011 to December 2020. Search results were screened by 2 independent reviewers first by title and abstract and then by full text. Disagreements were resolved through discussion with a third reviewer.

**Results:** 54 randomized controlled trials were included in our analysis. For induction of remission, azathioprine (OR, 3.5; 95% Crl, 1.4–8.9), infliximab (OR, 4.1; 95% Crl, 1.2–16.0), infliximab + azathioprine (OR, 7.0; 95% Crl, 1.2–41.0) and infliximab+ methotrexate (OR, 7.8; 95% Crl, 1.2–65.0) were more effective in first-line therapy than placebo. Adalimumab showed superiority to placebo in second-line therapy, but the range of SD was wide. For maintenance of remission, adalimumab (OR,2.24;95% Crl,1.17–4.76) and azathioprine (OR,2.05; 95% Crl,1.14–3.96) were more effective than placebo. Adalimumab (OR,0.56; 95%Crl,0.27–1.2), budesonide (OR,0.63; 95%Crl,0.26–1.6) and natalizumab (OR,0.65; 95%Crl,0.30–1.4) was associated with less risk of withdrawals when compared with placebo.

**Conclusion:** For induction of remission, azathioprine, infliximab, and infliximab + azathioprine were more effective in first-line therapy. In second-line therapy, adalimumab was more effective but should be interpreted carefully. For maintenance of remission, adalimumab and azathioprine were more effective. Besides, adalimumab, budesonide, natalizumab had lower withdrawals. Therefore, biological agents were not always better than non-biological agents and they have their own advantages in different treatment methods of Crohn's disease.

## Introduction

Crohn's disease (CD) is a chronic inflammatory disease of the gastrointestinal tract with symptoms evolving in a relapsing and remitting manner (1). It is also a progressive disease that leads to bowel damage and disability (1). Among adult CD patients, there is no particularly significant difference in the distribution of prevalence between men and women, and this disease usually occurs in young and middle-aged groups who are between 20 and 40 years old (2). The incidence of CD is always increasing in most parts of the world. The incidence and prevalence of CD is higher in developed countries than in developing countries, which is also higher in urban areas than in rural areas (2). It is reported that in addition to intestinal damage and disability, CD can also lead patients to experience symptoms of anxiety and/or depression, which will have a significant impact on quality of life (3). As the disease mechanism of CD remains unknown, a curative therapy is not yet available (4). The purpose of current treatment is to keep patients in remission. There are several drugs currently available for the treatment of CD, including non-biological agents: anti-inflammatory agents (such as mesalazine (5ASA), steroids [such as budesonide(BUD), prednisolone (PED)], immunosuppressive agents [such as azathioprine (AZA) and mercaptopurine (6MP), methotrexate (MTX)], and biologic therapies: anti-tumor necrosis factor (TNF) [such as infliximab (IFX), adalimumab (ADA), certolizumab pegol (CZP)], anti-α4β7 integrin [natalizumab (NTZ)], anti-alpha-4 integrin [vedolizumab(VDZ)] and anti-interleukin 12/23[ustekinumab (UST)] (5). However, the choice of treatments for induction and maintenance is still a challenge. Although there are currently many randomized clinical controlled trials (most of which are placebo-controlled) for traditional non-biological agents and those new biological agents (such as vedolizumab, ustekinumab), relevant head-to-head experiment comparison is few. The head-to-head comparison results can help making decisions, as well as providing clinicians and patients with medication reference.

Network Meta-analysis can help us conduct the integration of multiple clinical trials, especially in the absence of direct comparison evidence (6). Many previously published meta-analyses did not consider whether patients treated with biological agents had previously received treatment with anti-TNF agents, and the efficacy of patients treated with anti-TNF agents was significantly different from those who had not previously received treatment. Therefore, in our study, patients were divided into first-line treatment and second-line treatment according to whether they had received anti-TNF agents before. In addition, most of previously published researches (7–14) also focused on only biological agents, without considering the difference between the efficacy of traditional non-biological agents and biological agents. Some other researches (15, 16) only described the efficacy of immunosuppressants and didn't include some new biological agents. Therefore, based on the direct and indirect evidence in clinical trials, we conducted a network meta-analysis to compare the efficacy of therapies for induction and maintenance of remission including anti-inflammatory drugs, immunosuppressive agents, steroids, anti-TNF drugs, anti-α4β7 integrin, anti-alpha-4 integrin and anti-interleukin 12/23 or their combination in adult patients with CD.

## Methods

### Eligibility Criteria

We included all randomized controlled trials that assessed treatments (mesalazine, budesonide, azathioprine, sulfasalazine (SSZ), everolimus (EVE), olsalazine (OLS), mercaptopurine, methotrexate, infliximab, adalimumab, certolizumab pegol, vedolizumab, ustekinumab, natalizumab) alone or in combination in adult patients with CD. We included trials assessing the induction of remission of non-biological and biological agents between 2 and 18 weeks. We include trials assessing the maintenance of remission of remission with at least 24 weeks in duration.

Trials studying only pediatric or postoperative patients and those trials without fixed treatments were excluded (such as standard of care). In addition, studies exclusively assessing fistulizing CD, and those didn't report the clear remission as the outcome were also excluded.

The primary outcome was remission, which was defined as Crohn's Disease Activity Index (CDAI) <=150 or HBI (Harvey-Bradshaw index) <5. We chose the remission criteria defined in the research if the CDAI was not reported. Secondary end point was total withdrawals which was defined as the total number of patients who were withdrawn from the research after randomization for any reason. Eligibility criteria were established using the PICOS (see in the Supplementary Table 1). The Preferred Reporting Items for Systematic Reviews and Meta-Analyses guidelines were also followed (see in the Supplementary Table 2).

### Literature Search and Study Selection

We searched the relevant systematic reviews from January 2011 to July 2020, and selected the included trials that meet the eligibility criteria. Besides, we performed the database search through to December 2020 in MEDLINE by Ovid, Embase, and the Cochrane Library. The database and systematic reviews search strategies were reported in the Supplementary Table 3. Search results were screened by two independent reviewers first by title and abstract and then by full text. Disagreements were resolved through discussion with a third reviewer.

### Data Collection and Quality Appraisal

We extracted relevant characteristics from the relevant study. For induction, some articles reported results several time points, and we chose the result closest to 12 weeks as the primary outcome when the time point of the primary outcome was not specified or induction was not the primary goal of the study. For maintenance, we chose the time points closet to the end of the trial. Total withdrawals were extracted at the end of the trials for both induction and maintenance trials. Baseline disease severity was defined as CDAI (220-450) or HBI > 7.

We extracted the number of patients after randomization and those who experienced the outcome. If the outcomes were only reported in the graphic format, the software Engauge Digitizer 12.0 was used to get the percentages and the number would be calculated and rounded to the nearest whole number. The quality of trials was rated through the Cochrane Risk of Bias tool (17).

### Synthesis of Results

We used a random-effects Bayesian network Meta-analysis to research treatment effects for remission and total withdrawals. For the clinical heterogeneity across trials, the random-effects model was more appropriate. R statistical software version 3.6.0 was used to do the statistical analyses with the gemtc package version 0.8-2 (www.r-project.org) and do the funnel plots with the netmeta package. The risk of bias graph was generated by Cochrane RevMan 5.3.

We chose uninformative prior probability distribution for all variables, each model uses 4 Markov chains to set the initial value, and the number of iterations is set to 20,000.

### Sensitivity Analyses

In order to assess the robustness of the results, we did several sensitivity analyses for induction of remission, which was following: excluding trials with a high risk of bias; For maintenance of remission, the additional sensitivity analyses were following: including trials whose time points were 1 year or longer; For the withdrawal, the sensitivity analysis was following: including those withdrawal because of the adverse events.

## Results

**Figure 1:**
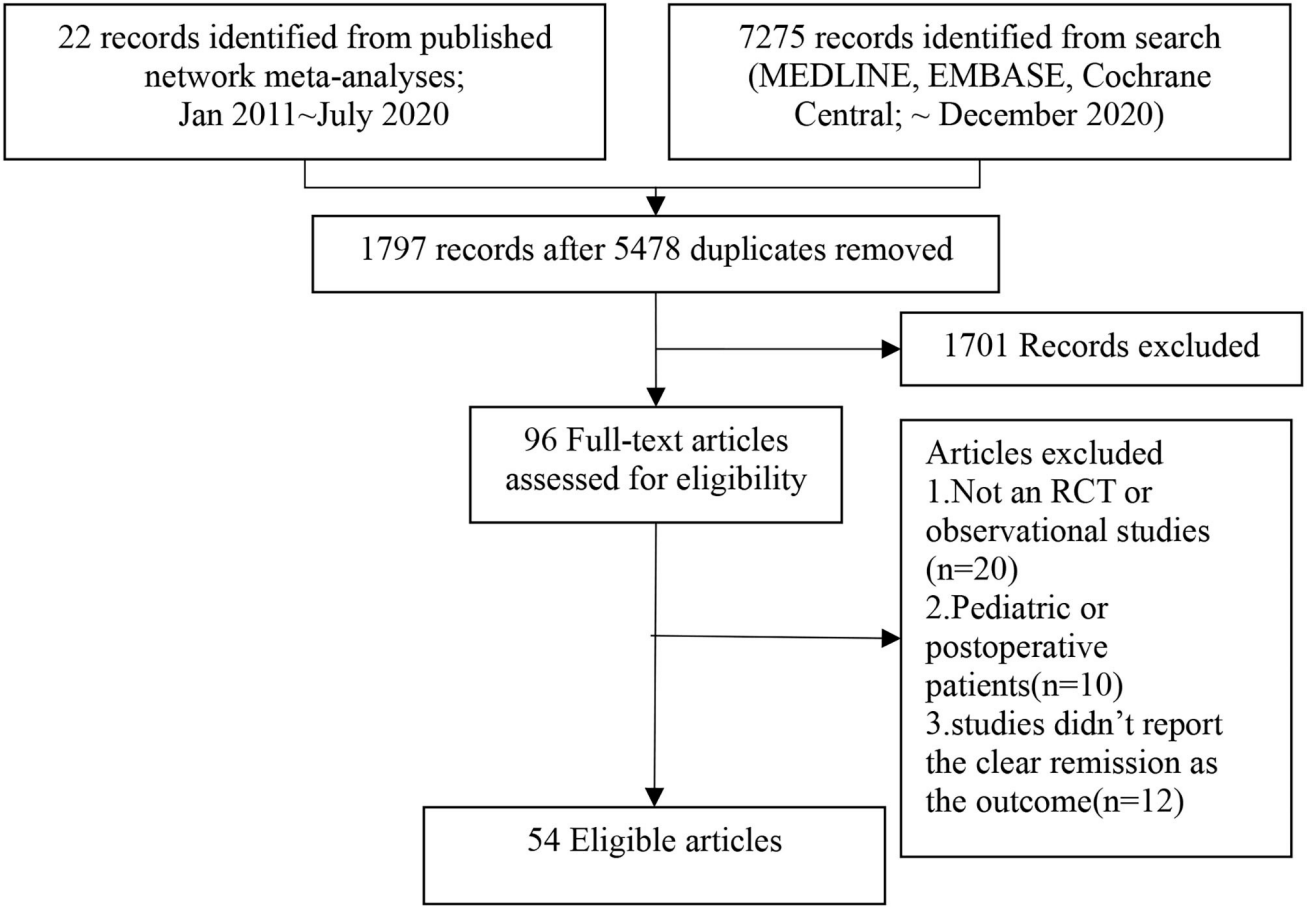
Flow chat of eligible studies selection procedures.

Finally, we included 54 studies. PRISMA flow diagram could be found in Figure 1. Characteristics of included trials were shown in Supplementary Data Sheet 1. There were 6 trials (18–23) evaluated adalimumab, 4 trials (24–27) evaluated infliximab, 4 trials (28–31) evaluated certolizumab, 3 trials (32–34) evaluated vedolizumab, 1 trial (35) evaluated ustekinumab, 5 trials (36–40) evaluated natalizumab, 9 trials (41–49) evaluated immunosuppressants, 7 trials (50–56) evaluated anti-inflammatory drugs, 9 trials (57–65) evaluated glucocorticoid, 6 trials (66–71) evaluated combination therapy. Forty three trials provided data on induction of remission and 19 trials provided data on maintenance of remission. 46 trials provided the information about the concomitant therapy, which was reported in the Supplementary Data Sheet 1. CDAI was used to define remission in most trials except 1 trial (25) which used HBI. The risk of bias was judged to be high in the 7 trials (37, 38, 45, 46, 50, 53, 66). All these 7 trials evaluated the induction of remission and they were excluded in the sensitivity analyses. Model structures were shown in the Supplementary Table 4. A detailed assessment of the risk of bias was presented in Supplementary Table 5 and Supplementary Data Sheet 2.

To assess the consistency of the evidence, we used the node-splitting analysis to study whether the results could be jointly summarized and plotted (72). The node-splitting analysis would produce the results by the direct comparison and indirect comparison. If there was no difference between their results, they were consistency. This function could be available by the mtc.nodesplit command in the gemtc package.

### Synthesis of Results

#### Induction of Remission

##### First-Line Therapy for CD

**Figure 2:**
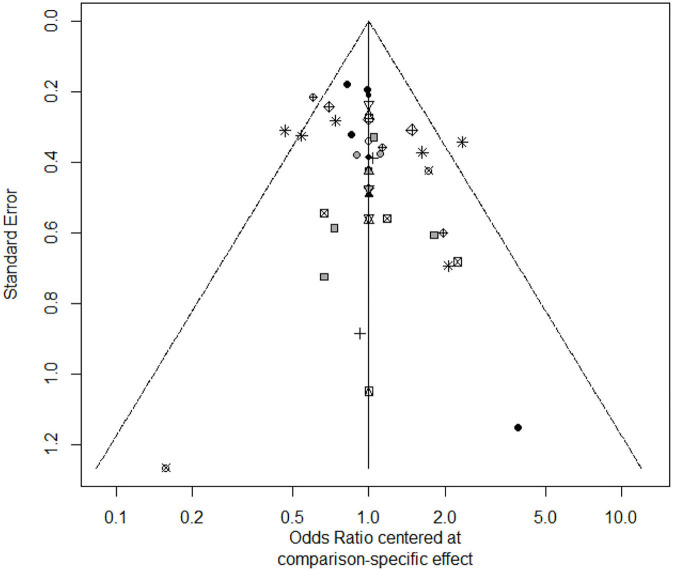
The funnel plot of first-line therapy for induction of remission.

For the patients who did not receive anti-TNF agents, AZA, IFX, IFX+AZA, IFX+MTX were different from placebo for inducing remission, which could be seen in the Table 1. AZA, IFX, IFX+AZA, IFX+MTX had a 1.6, 1.8, 30.8, and 39.3% cumulative probability of ranking highest for induction of remission (Supplementary Table 6). The funnel plot showed no significant asymmetry in the Figure 2.

**Table 1:** First-line therapy network meta-analysis results for induction of remission.

	**5ASA**	**X6MP**	**ADA**	**ADAAZA**	**AZA**	**BUD**	**CZP**	**EVE**	**IFX**	**IFXAZA**	**IFXMTX**	**MTX**	**NTZ**	**OLS**	**P**	**SSZ**	**SSZ6MP**	**UST**	**VDZ**
5ASA	5ASA	−0.13 (-1.59, 1.26)	−0.06 (-1.49, 1.41)	−0.8 (-3.08, 1.47)	0.65 (-0.58, 1.84)	−0.06 (-0.92, 0.79)	0.26 (-1.04, 1.58)	−0.59 (-2.5, 1.29)	0.80 (-0.66, 2.3)	1.32 (-0.59, 3.23)	1.43 (-0.63, 3.66)	0.44 (-1.6, 2.45)	0.04 (-1.15, 1.25)	**-2.17 (-4.28,−0.11)**	−0.61 (-1.41, 0.15)	−0.85 (-2.72, 0.92)	0.47 (-1.43, 2.29)	0.18 (-1.71, 2.02)	0.17 (-1.35, 1.63)
6MP	0.13 (-1.26, 1.59)	6MP	0.07 (-1.75, 1.99)	−0.67 (-3.2, 1.94)	0.79 (-0.88, 2.47)	0.07 (-1.41, 1.61)	0.39 (-1.32, 2.18)	−0.45 (-2.67, 1.78)	0.93 (-0.9, 2.87)	1.46 (-0.76, 3.71)	1.56 (-0.77, 4.11)	0.57 (-1.77, 2.93)	0.17 (-1.46, 1.9)	−2.04 (-4.42, 0.35)	−0.48 (-1.87, 0.93)	−0.72 (-2.49, 1.01)	0.6 (-1.2, 2.39)	0.31 (-1.88, 2.54)	0.3 (-1.59, 2.21)
ADA	0.06 (-1.41, 1.49)	−0.07 (-1.99, 1.75)	ADA	−0.74 (-2.52, 1.04)	0.72 (-0.75, 2.1)	0 (-1.42, 1.37)	0.32 (-1.31, 1.92)	−0.53 (-2.66, 1.52)	0.86 (-0.47, 2.2)	1.38 (-0.57, 3.31)	1.49 (-0.47, 3.6)	0.5 (-1.76, 2.7)	0.1 (-1.44, 1.63)	−2.11 (-4.43, 0.13)	−0.55 (-1.81, 0.65)	−0.79 (-2.98, 1.27)	0.52 (-1.67, 2.61)	0.23 (-1.88, 2.3)	0.23 (-1.57, 1.95)
ADAAZA	0.8 (-1.47, 3.08)	0.67 (-1.94, 3.2)	0.74 (-1.04, 2.52)	ADAAZA	1.45 (-0.84, 3.69)	0.74 (-1.52, 2.97)	1.06 (-1.34, 3.44)	0.22 (-2.54, 2.91)	1.59 (-0.6, 3.83)	2.12 (-0.51, 4.72)	2.23 (-0.4, 5.01)	1.24 (-1.62, 4.05)	0.85 (-1.5, 3.16)	−1.37 (-4.29, 1.49)	0.19 (-1.98, 2.32)	−0.06 (-2.88, 2.64)	1.27 (-1.57, 4)	0.97 (-1.79, 3.7)	0.96 (-1.57, 3.43)
AZA	−0.65 (-1.84, 0.58)	−0.79 (-2.47, 0.88)	−0.72 (-2.1, 0.75)	−1.45 (-3.69, 0.84)	AZA	−0.71 (-1.85, 0.44)	−0.4 (-1.78, 1.04)	−1.24 (-2.96, 0.48)	0.14 (-1.1, 1.48)	0.67 (-0.92, 2.31)	0.78 (-1.15, 2.89)	−0.21 (-2.29, 1.88)	−0.61 (-1.9, 0.72)	**-2.82 (-4.97,−0.69)**	**-1.26 (-2.19,−0.33)**	−1.51 (-3.47, 0.42)	−0.19 (-2.18, 1.78)	−0.48 (-2.39, 1.46)	−0.49 (-2.05, 1.09)
BUD	0.06 (-0.79, 0.92)	−0.07 (-1.61, 1.41)	0 (-1.37, 1.42)	−0.74 (-2.97, 1.52)	0.71 (-0.44, 1.85)	BUD	0.31 (-0.92, 1.57)	−0.53 (-2.41, 1.32)	0.85 (-0.54, 2.32)	1.38 (-0.46, 3.25)	1.49 (-0.52, 3.69)	0.5 (-1.49, 2.48)	0.1 (-1.02, 1.25)	**-2.11 (-4.17,−0.1)**	−0.55 (-1.23, 0.11)	−0.8 (-2.66, 0.98)	0.52 (-1.37, 2.37)	0.24 (-1.59, 2.05)	0.22 (-1.23, 1.65)
CZP	−0.26 (-1.58, 1.04)	−0.39 (-2.18, 1.32)	−0.32 (-1.92, 1.31)	−1.06 (-3.44, 1.34)	0.4 (-1.04, 1.78)	−0.31 (-1.57, 0.92)	CZP	−0.84 (-2.9, 1.16)	0.54 (-1.08, 2.2)	1.07 (-0.98, 3.09)	1.17 (-1, 3.51)	0.18 (-1.97, 2.29)	−0.21 (-1.61, 1.19)	**-2.43 (-4.64,−0.27)**	−0.86 (-1.93, 0.16)	−1.12 (-3.18, 0.84)	0.21 (-1.88, 2.24)	−0.08 (-2.1, 1.9)	−0.09 (-1.75, 1.54)
EVE	0.59 (-1.29, 2.5)	0.45 (-1.78, 2.67)	0.53 (-1.52, 2.66)	−0.22 (-2.91, 2.54)	1.24 (-0.48, 2.96)	0.53 (-1.32, 2.41)	0.84 (-1.16, 2.9)	EVE	1.38 (-0.62, 3.47)	1.91 (-0.39, 4.26)	2.02 (-0.47, 4.67)	1.03 (-1.51, 3.58)	0.63 (-1.3, 2.64)	−1.58 (-4.19, 1)	−0.02 (-1.75, 1.72)	−0.28 (-2.74, 2.17)	1.06 (-1.46, 3.52)	0.76 (-1.65, 3.21)	0.75 (-1.4, 2.91)
IFX	−0.8 (-2.3, 0.66)	−0.93 (-2.87, 0.9)	−0.86 (-2.2, 0.47)	−1.59 (-3.83, 0.6)	−0.14 (-1.48, 1.1)	−0.85 (-2.32, 0.54)	−0.54 (-2.2, 1.08)	−1.38 (-3.47, 0.62)	IFX	0.53 (-1.12, 2.12)	0.63 (-0.85, 2.25)	−0.36 (-2.64, 1.85)	−0.75 (-2.32, 0.79)	**-2.97 (-5.31,−0.71)**	**-1.4 (-2.72,−0.18)**	−1.65 (-3.86, 0.42)	−0.33 (-2.56, 1.78)	−0.62 (-2.75, 1.45)	−0.63 (-2.47, 1.11)
IFXAZA	−1.32 (-3.23, 0.59)	−1.46 (-3.71, 0.76)	−1.38 (-3.31, 0.57)	−2.12 (-4.72, 0.51)	−0.67 (-2.31, 0.92)	−1.38 (-3.25, 0.46)	−1.07 (-3.09, 0.98)	−1.91 (-4.26, 0.39)	−0.53 (-2.12, 1.12)	IFXAZA	0.1 (-2.07, 2.45)	−0.88 (-3.45, 1.65)	−1.28 (-3.25, 0.7)	**-3.49 (-6.1,−0.92)**	**-1.93 (-3.68,−0.21)**	−2.19 (-4.67, 0.23)	−0.86 (-3.38, 1.57)	−1.15 (-3.6, 1.27)	−1.16 (-3.34, 0.96)
IFXMTX	−1.43 (-3.66, 0.63)	−1.56 (-4.11, 0.77)	−1.49 (-3.6, 0.47)	−2.23 (-5.01, 0.4)	−0.78 (-2.89, 1.15)	−1.49 (-3.69, 0.52)	−1.17 (-3.51, 1)	−2.02 (-4.67, 0.47)	−0.63 (-2.25, 0.85)	−0.1 (-2.45, 2.07)	IFXMTX	−0.99 (-3.8, 1.66)	−1.38 (-3.66, 0.74)	**-3.6 (-6.46,−0.95)**	**-2.04 (-4.15,−0.13)**	−2.29 (-5.06, 0.23)	−0.97 (-3.75, 1.59)	−1.25 (-3.99, 1.27)	−1.26 (-3.76, 1)
MTX	−0.44 (-2.45, 1.6)	−0.57 (-2.93, 1.77)	−0.5 (-2.7, 1.76)	−1.24 (-4.05, 1.62)	0.21 (-1.88, 2.29)	−0.5 (-2.48, 1.49)	−0.18 (-2.29, 1.97)	−1.03 (-3.58, 1.51)	0.36 (-1.85, 2.64)	0.88 (-1.65, 3.45)	0.99 (-1.66, 3.8)	MTX	−0.4 (-2.46, 1.71)	−2.61 (-5.3, 0.06)	−1.05 (-2.92, 0.81)	−1.3 (-3.88, 1.21)	0.03 (-2.57, 2.58)	−0.26 (-2.8, 2.25)	−0.28 (-2.52, 1.98)
NTZ	−0.04 (-1.25, 1.15)	−0.17 (-1.9, 1.46)	−0.1 (-1.63, 1.44)	−0.85 (-3.16, 1.5)	0.61 (-0.72, 1.9)	−0.1 (-1.25, 1.02)	0.21 (-1.19, 1.61)	−0.63 (-2.64, 1.3)	0.75 (-0.79, 2.32)	1.28 (-0.7, 3.25)	1.38 (-0.74, 3.66)	0.4 (-1.71, 2.46)	NTZ	**-2.21 (-4.38,−0.12)**	−0.65 (-1.6, 0.23)	−0.9 (-2.9, 1)	0.42 (-1.6, 2.37)	0.14 (-1.83, 2.04)	0.12 (-1.47, 1.67)
OLS	**2.17 (0.11, 4.28)**	2.04 (-0.35, 4.42)	2.11 (-0.13, 4.43)	1.37 (-1.49, 4.29)	**2.82 (0.69, 4.97)**	**2.11 (0.1, 4.17)**	**2.43 (0.27, 4.64)**	1.58 (-1, 4.19)	**2.97 (0.71, 5.31)**	**3.49 (0.92, 6.1)**	**3.6 (0.95, 6.46)**	2.61 (-0.06, 5.3)	**2.21 (0.12, 4.38)**	OLS	1.56 (-0.35, 3.5)	1.32 (-1.31, 3.89)	2.64 (0.02, 5.23)	2.35 (-0.2, 4.92)	2.34 (0.03, 4.63)
P	0.61 (-0.15, 1.41)	0.48 (-0.93, 1.87)	0.55 (-0.65, 1.81)	−0.19 (-2.32, 1.98)	**1.26 (0.33, 2.19)**	0.55 (-0.11, 1.23)	0.86 (-0.16, 1.93)	0.02 (-1.72, 1.75)	**1.4 (0.18, 2.72)**	**1.93 (0.21, 3.68)**	**2.04 (0.13, 4.15)**	1.05 (-0.81, 2.92)	0.65 (-0.23, 1.6)	−1.56 (-3.5, 0.35)	P	−0.25 (-1.99, 1.44)	1.07 (-0.71, 2.82)	0.79 (-0.91, 2.48)	0.77 (-0.5, 2.05)
SSZ	0.85 (-0.92, 2.72)	0.72 (-1.01, 2.49)	0.79 (-1.27, 2.98)	0.06 (-2.64, 2.88)	1.51 (-0.42, 3.47)	0.8 (-0.98, 2.66)	1.12 (-0.84, 3.18)	0.28 (-2.17, 2.74)	1.65 (-0.42, 3.86)	2.19 (-0.23, 4.67)	2.29 (-0.23, 5.06)	1.3 (-1.21, 3.88)	0.9 (-1, 2.9)	−1.32 (-3.89, 1.31)	0.25 (-1.44, 1.99)	SSZ	1.32 (-0.51, 3.18)	1.03 (-1.36, 3.47)	1.03 (-1.09, 3.2)
SSZ6MP	−0.47 (-2.29, 1.43)	−0.6 (-2.39, 1.2)	−0.52 (-2.61, 1.67)	−1.27 (-4, 1.57)	0.19 (-1.78, 2.18)	−0.52 (-2.37, 1.37)	−0.21 (-2.24, 1.88)	−1.06 (-3.52, 1.46)	0.33 (-1.78, 2.56)	0.86 (-1.57, 3.38)	0.97 (-1.59, 3.75)	−0.03 (-2.58, 2.57)	−0.42 (-2.37, 1.6)	−2.64 (-5.23,−0.02)	−1.07 (-2.82, 0.71)	−1.32 (-3.18, 0.51)	SSZ6MP	**-0.29 (-2.73, 2.19)**	−0.3 (-2.45, 1.9)
UST	−0.18 (-2.02, 1.71)	−0.31 (-2.54, 1.88)	−0.23 (-2.3, 1.88)	−0.97 (-3.7, 1.79)	0.48 (-1.46, 2.39)	−0.24 (-2.05, 1.59)	0.08 (-1.9, 2.1)	−0.76 (-3.21, 1.65)	0.62 (-1.45, 2.75)	1.15 (-1.27, 3.6)	1.25 (-1.27, 3.99)	0.26 (-2.25, 2.8)	−0.14 (-2.04, 1.83)	−2.35 (-4.92, 0.2)	−0.79 (-2.48, 0.91)	−1.03 (-3.47, 1.36)	**0.29 (-2.19, 2.73)**	UST	−0.01 (-2.15, 2.12)
VDZ	−0.17 (-1.63, 1.35)	−0.3 (-2.21, 1.59)	−0.23 (-1.95, 1.57)	−0.96 (-3.43, 1.57)	0.49 (-1.09, 2.05)	−0.22 (-1.65, 1.23)	0.09 (-1.54, 1.75)	−0.75 (-2.91, 1.4)	0.63 (-1.11, 2.47)	1.16 (-0.96, 3.34)	1.26 (-1, 3.76)	0.28 (-1.98, 2.52)	−0.12 (-1.67, 1.47)	−2.34 (-4.63,−0.03)	−0.77 (-2.05, 0.5)	−1.03 (-3.2, 1.09)	0.3 (-1.9, 2.45)	0.01 (-2.12, 2.15)	VDZ

*5ASA, mesalazine; BUD, budesonide; AZA, azathioprine; 6MP, mercaptopurine; MTX, methotrexate; IFX, infliximab; ADA, adalimumab; CZP, certolizumab pegol; NTZ, natalizumab; VDZ, vedolizumab; UST, ustekinumab; SSZ, sulfasalazine; EVE, everolimus; OLS, olsalazine; P, Placebo. The bold values meant that they were significantly different from the placebo*.

In the sensitivity analyses, when we excluded those trials with high bias risk, we could find that AZA, IFX, IFX + AZA were different from placebo for inducing remission, which was reported in the Supplementary Table 7.

The node-splitting analysis of inconsistency for induction of remission was reported in the Supplementary Table 8.

##### Second-Line Therapy for CD

For the patients who received anti-TNF agents, ADA, NTZ, UST, VDZ were not different form placebo for inducing remission, which could be found in the Table 2, but we could find that the ranges of SD were large, so the reliability of point estimates were low. Instead, the probability of ranking was more credible. We could find that ADA (60%) and UST (21%) might have a higher probability of ranking highest for induction of remission (Supplementary Table 9).

**Table 2:** Second-line therapy network meta-analysis results for induction of remission.

	**ADA**	**NTZ**	**P**	**UST**	**VDZ**
ADA	ADA	−0.84 (−2.69, 0.96)	−1.12 (−2.34, −0.01)	−0.47 (−2.24, 1.23)	−0.94 (−2.73, 0.79)
NTZ	0.84 (−0.96, 2.69)	NTZ	−0.28 (−1.7, 1.1)	0.37 (−1.55, 2.29)	−0.1 (−2.02, 1.81)
P	1.12 (0.01, 2.34)	0.28 (−1.1, 1.7)	P	0.64 (−0.65, 1.95)	0.18 (−1.14, 1.51)
UST	0.47 (−1.23, 2.24)	−0.37 (−2.29, 1.55)	−0.64 (−1.95, 0.65)	UST	−0.46 (−2.32, 1.37)
VDZ	0.94 (−0.79, 2.73)	0.10 (−1.81, 2.02)	−0.18 (−1.51, 1.14)	0.46 (−1.37, 2.32)	VDZ

*ADA, adalimumab; NTZ, natalizumab; VDZ, vedolizumab; UST, ustekinumab; P, placebo*.

#### Maintenance of Remission

**Figure 3:**
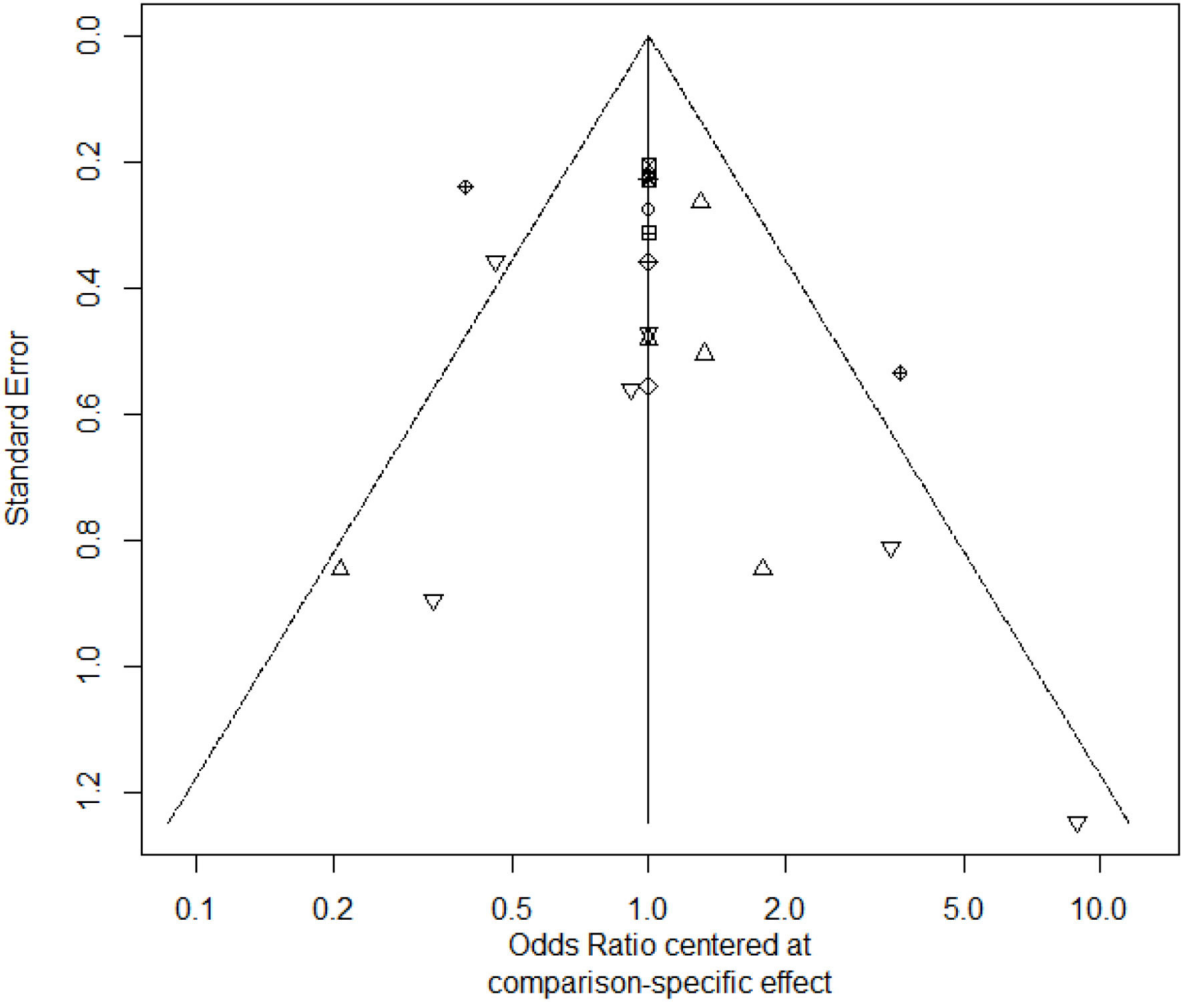
The funnel plot of maintenance of remission.

ADA and AZA were different form placebo for maintenance of remission, and other treatments were not different from placebo, which could be found in the Table 3. Rank probability for maintenance of remission was shown in the Supplementary Table 10. The funnel plot showed no significant asymmetry in the Figure 3.

**Table 3:** Network meta-analysis results for maintenance of remission.

	**ADA**	**AZA**	**CZP**	**IFX**	**IFXAZA**	**IFXMTX**	**MTX**	**NTZ**	**P**	**UST**	**VDZ**
ADA	ADA	−0.09 (−1.01, 0.81)	−0.28 (−1.9, 1.23)	−0.43 (−1.35, 0.45)	0.18 (−1.29, 1.61)	−0.41 (−2.1, 1.23)	−0.26 (−1.56, 0.99)	0.16 (−1.48, 1.73)	–**0.81 (**–**1.56**, –**0.15)**	−0.46 (−2.08, 1.06)	−0.26 (−1.89, 1.26)
AZA	0.09 (−0.81, 1.01)	AZA	−0.19 (−1.77, 1.3)	−0.34 (−1.22, 0.52)	0.27 (−1.03, 1.55)	−0.33 (−1.99, 1.33)	−0.17 (−1.27, 0.89)	0.25 (−1.35, 1.81)	–**0.72 (**–**1.38**, –**0.14)**	−0.37 (−1.94, 1.11)	−0.17 (−1.75, 1.33)
CZP	0.28 (−1.23, 1.9)	0.19 (−1.3, 1.77)	CZP	−0.15 (−1.7, 1.48)	0.46 (−1.43, 2.42)	−0.13 (−2.24, 2.05)	0.02 (−1.71, 1.81)	0.45 (−1.55, 2.45)	−0.53 (−1.91, 0.88)	−0.18 (−2.15, 1.8)	0.02 (−1.95, 2.01)
IFX	0.43 (−0.45, 1.35)	0.34 (−0.52, 1.22)	0.15 (−1.48, 1.7)	IFX	0.61 (−0.68, 1.9)	0.01 (−1.39, 1.43)	0.18 (−1.12, 1.44)	0.6 (−1.08, 2.2)	−0.38 (−1.17, 0.36)	−0.03 (−1.66, 1.54)	0.17 (−1.47, 1.74)
IFXAZA	−0.18 (−1.61, 1.29)	−0.27 (−1.55, 1.03)	−0.46 (−2.42, 1.43)	−0.61 (−1.9, 0.68)	IFXAZA	−0.59 (−2.49, 1.32)	−0.43 (−2.08, 1.19)	−0.01 (−2.01, 1.93)	−0.98 (−2.34, 0.31)	−0.64 (−2.6, 1.26)	−0.44 (−2.42, 1.46)
IFXMTX	0.41 (−1.23, 2.1)	0.33 (−1.33, 1.99)	0.13 (−2.05, 2.24)	−0.01 (−1.43, 1.39)	0.59 (−1.32, 2.49)	IFXMTX	0.16 (−1.77, 2.04)	0.58 (−1.6, 2.71)	−0.39 (−2.02, 1.18)	−0.04 (−2.22, 2.05)	0.15 (−2.03, 2.27)
MTX	0.26 (−0.99, 1.56)	0.17 (−0.89, 1.27)	−0.02 (−1.81, 1.71)	−0.18 (−1.44, 1.12)	0.43 (−1.19, 2.08)	−0.16 (−2.04, 1.77)	MTX	0.42 (−1.39, 2.21)	−0.55 (−1.64, 0.52)	−0.21 (−1.99, 1.55)	−0.01 (−1.79, 1.76)
NTZ	−0.16 (−1.73, 1.48)	−0.25 (−1.81, 1.35)	−0.45 (−2.45, 1.55)	−0.6 (−2.2, 1.08)	0.01 (−1.93, 2.01)	−0.58 (−2.71, 1.6)	−0.42 (−2.21, 1.39)	NTZ	−0.97 (−2.43, 0.47)	−0.63 (−2.64, 1.38)	−0.43 (−2.45, 1.59)
P	**0.81 (0.15, 1.56)**	**0.72 (0.14, 1.38)**	0.53 (−0.88, 1.91)	0.38 (−0.36, 1.17)	0.98 (−0.31, 2.34)	0.39 (−1.18, 2.02)	0.55 (−0.52, 1.64)	0.97 (−0.47, 2.43)	P	0.35 (−1.06, 1.74)	0.55 (−0.87, 1.95)
UST	0.46 (−1.06, 2.08)	0.37 (−1.11, 1.94)	0.18 (−1.8, 2.15)	0.03 (−1.54, 1.66)	0.64 (−1.26, 2.6)	0.04 (−2.05, 2.22)	0.21 (−1.55, 1.99)	0.63 (−1.38, 2.64)	−0.35 (−1.74, 1.06)	UST	0.2 (−1.78, 2.2)
VDZ	0.26 (−1.26, 1.89)	0.17 (−1.33, 1.75)	−0.02 (−2.01, 1.95)	−0.17 (−1.74, 1.47)	0.44 (−1.46, 2.42)	−0.15 (−2.27, 2.03)	0.01 (−1.76, 1.79)	0.43 (−1.59, 2.45)	−0.55 (−1.95, 0.87)	−0.2 (−2.2, 1.78)	VDZ

*AZA, azathioprine; MTX, methotrexate; IFX, infliximab; ADA, adalimumab; CZP, certolizumab pegol; NTZ, natalizumab; VDZ, vedolizumab; UST, ustekinumab; P, Placebo. The bold values meant that they were significantly different from the placebo*.

The node-splitting analysis of inconsistency for maintenance of remission was reported in the Supplementary Table 11.

In the sensitivity analyses, when we included those trials whose time points were 1 year or longer, we could find that ADA and AZA were different form placebo for maintenance of remission, which was reported in the Supplementary Table 12.

### Withdrawals

**Figure 4:**
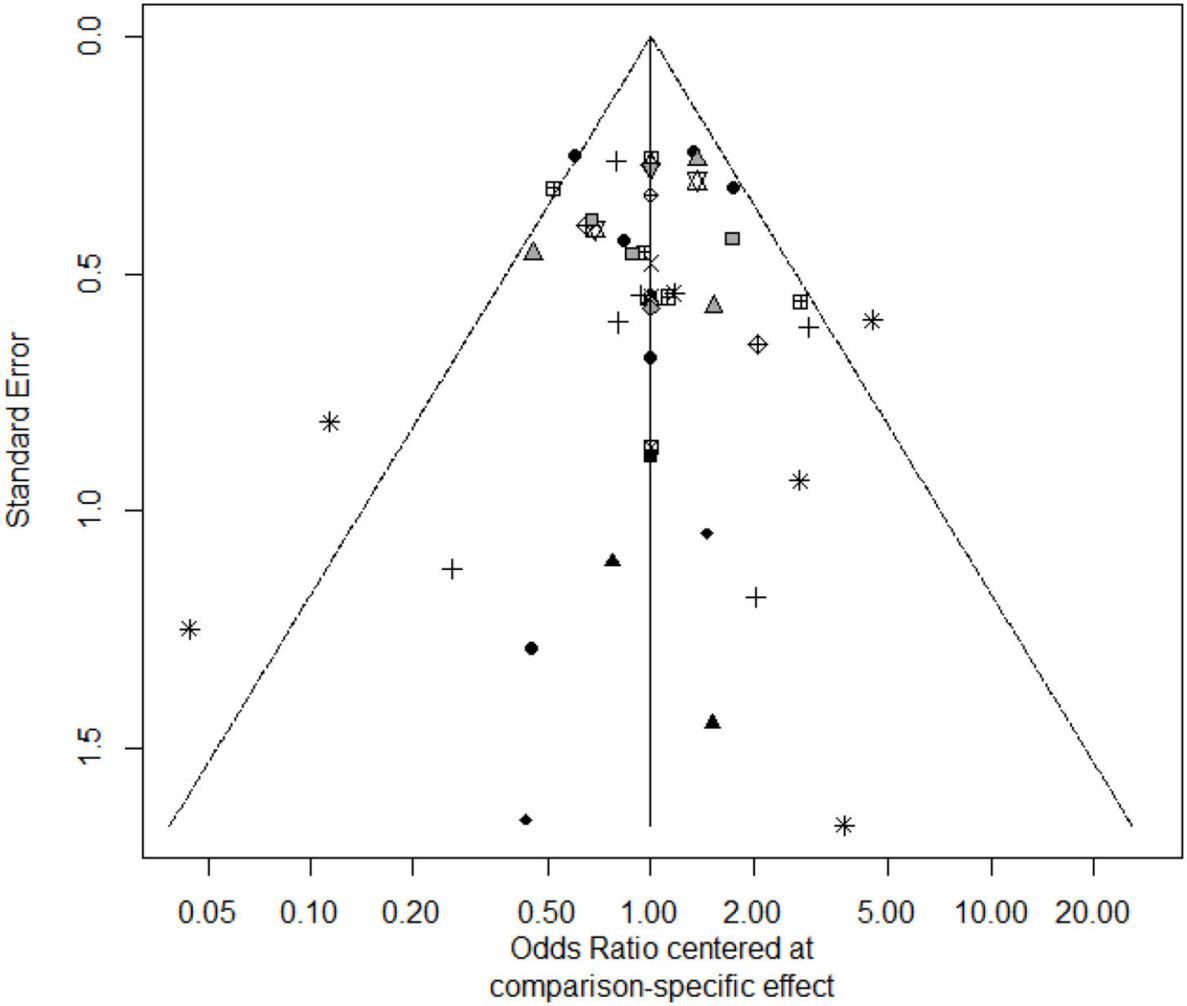
The funnel plot of withdrawals.

For total withdrawals, ADA, BUD and NTZ were not different form placebo, because their 95% confidence intervals were wide, which could be seen in the Table 4. 6-MP and MTX were associated with more WDAEs than placebo, which could be seen in the Supplementary Table 13. The funnel plot showed no significant asymmetry in the Figure 4.

**Table 4:** Network meta-analysis results for withdrawals.

	**5ASA**	**6MP**	**ADA**	**ADAAZA**	**AZA**	**BUD**	**CZP**	**EVE**	**IFX**	**IFXAZA**	**MTX**	**NTZ**	**NTZIFX**	**P**	**PED**	**SSZ**	**SSZ6MP**	**UST**	**VDZ**
5ASA	5ASA	0.59 (−0.63, 1.81)	−0.84 (−1.89, 0.23)	−0.54 (−2.42, 1.37)	−0.21 (−1.3, 0.88)	−0.73 (−1.56, 0.15)	−0.06 (−1.19, 1.11)	0.44 (−1.3, 2.2)	0.95 (−0.25, 2.31)	0.45 (−1.2, 2.2)	0.81 (−0.68, 2.37)	−0.68 (−1.78, 0.38)	0.88 (−1.39, 3.32)	−0.25 (−1.04, 0.52)	−0.47 (−1.88, 1.03)	0.2 (−1.38, 1.75)	0.6 (−0.97, 2.16)	−0.5 (−1.74, 0.73)	−0.32 (−2.3, 1.71)
6MP	−0.59 (−1.81, 0.63)	6MP	−1.43 (−2.82, −0.02)	−1.13 (−3.23, 1)	−0.8 (−2.22, 0.62)	−1.32 (−2.7, 0.1)	−0.65 (−2.11, 0.84)	−0.15 (−2.11, 1.82)	0.36 (−1.13, 2)	−0.14 (−2.02, 1.82)	0.22 (−1.52, 2.02)	−1.27 (−2.71, 0.12)	0.29 (−2.17, 2.88)	−0.85 (−2.05, 0.34)	−1.07 (−2.87, 0.85)	−0.39 (−1.86, 1.05)	0.01 (−1.45, 1.45)	−1.09 (−2.63, 0.42)	−0.9 (−3.08, 1.29)
ADA	0.84 (−0.23, 1.89)	1.43 (0.02, 2.82)	ADA	0.3 (−1.29, 1.88)	0.63 (−0.42, 1.65)	0.11 (−1.07, 1.29)	0.78 (−0.34, 1.9)	1.29 (−0.44, 2.99)	1.79 (0.72, 2.98)	1.29 (−0.3, 2.94)	1.65 (0.19, 3.15)	0.16 (−0.92, 1.18)	1.72 (−0.5, 4.08)	0.58 (−0.17, 1.3)	0.37 (−1.31, 2.11)	1.04 (−0.62, 2.63)	1.44 (−0.21, 3.05)	0.34 (−0.89, 1.53)	0.53 (−1.45, 2.52)
ADAAZA	0.54 (−1.37, 2.42)	1.13 (−1, 3.23)	−0.3 (−1.88, 1.29)	ADAAZA	0.33 (−1.57, 2.2)	−0.18 (−2.17, 1.78)	0.48 (−1.46, 2.41)	0.98 (−1.35, 3.29)	1.48 (−0.4, 3.5)	0.99 (−1.25, 3.27)	1.35 (−0.78, 3.53)	−0.14 (−2.07, 1.71)	1.41 (−1.3, 4.25)	0.28 (−1.48, 2)	0.07 (−2.23, 2.4)	0.75 (−1.56, 2.98)	1.14 (−1.17, 3.4)	0.04 (−1.98, 2)	0.22 (−2.3, 2.75)
AZA	0.21 (−0.88, 1.3)	0.8 (−0.62, 2.22)	−0.63 (−1.65, 0.42)	−0.33 (−2.2, 1.57)	AZA	−0.52 (−1.71, 0.7)	0.15 (−0.98, 1.32)	0.65 (−0.9, 2.21)	1.15 (0.18, 2.3)	0.66 (−0.72, 2.14)	1.02 (−0.29, 2.41)	−0.47 (−1.57, 0.59)	1.08 (−1.09, 3.43)	−0.05 (−0.82, 0.72)	−0.27 (−1.93, 1.52)	0.41 (−1.24, 2.04)	0.8 (−0.84, 2.46)	−0.29 (−1.54, 0.93)	−0.11 (−2.09, 1.92)
BUD	0.73 (−0.15, 1.56)	1.32 (−0.1, 2.7)	−0.11 (−1.29, 1.07)	0.18 (−1.78, 2.17)	0.52 (−0.7, 1.71)	BUD	0.67 (−0.58, 1.91)	1.17 (−0.66, 2.97)	1.67 (0.37, 3.1)	1.18 (−0.56, 2.97)	1.54 (−0.05, 3.16)	0.05 (−1.19, 1.19)	1.61 (−0.74, 4.08)	0.47 (−0.48, 1.37)	0.25 (−1.06, 1.65)	0.93 (−0.79, 2.56)	1.33 (−0.37, 2.98)	0.23 (−1.13, 1.54)	0.41 (−1.64, 2.48)
CZP	0.06 (−1.11, 1.19)	0.65 (−0.84, 2.11)	−0.78 (−1.9, 0.34)	−0.48 (−2.41, 1.46)	−0.15 (−1.32, 0.98)	−0.67 (−1.91, 0.58)	CZP	0.5 (−1.3, 2.28)	1.01 (−0.26, 2.38)	0.51 (−1.19, 2.27)	0.87 (−0.67, 2.45)	−0.62 (−1.8, 0.48)	0.94 (−1.38, 3.39)	−0.19 (−1.07, 0.63)	−0.41 (−2.13, 1.38)	0.26 (−1.45, 1.9)	0.66 (−1.04, 2.32)	−0.44 (−1.74, 0.82)	−0.26 (−2.28, 1.8)
EVE	−0.44 (−2.2, 1.3)	0.15 (−1.82, 2.11)	−1.29 (−2.99, 0.44)	−0.98 (−3.29, 1.35)	−0.65 (−2.21, 0.9)	−1.17 (−2.97, 0.66)	−0.5 (−2.28, 1.3)	EVE	0.5 (−1.22, 2.36)	0.01 (−2.01, 2.11)	0.37 (−1.58, 2.4)	−1.13 (−2.88, 0.6)	0.43 (−2.17, 3.19)	−0.7 (−2.28, 0.86)	−0.92 (−3.07, 1.33)	−0.24 (−2.4, 1.88)	0.16 (−2, 2.3)	−0.95 (−2.79, 0.88)	−0.76 (−3.18, 1.66)
IFX	−0.95 (−2.31, 0.25)	−0.36 (−2, 1.13)	−1.79 (−2.98, −0.72)	−1.48 (−3.5, 0.4)	−1.15 (−2.3, −0.18)	−1.67 (−3.1, −0.37)	−1.01 (−2.38, 0.26)	−0.5 (−2.36, 1.22)	IFX	−0.49 (−1.96, 0.88)	−0.14 (−1.58, 1.24)	−1.63 (−3, −0.45)	−0.08 (−2.03, 1.96)	−1.2 (−2.3, −0.27)	−1.42 (−3.28, 0.4)	−0.74 (−2.62, 0.94)	−0.35 (−2.21, 1.36)	−1.45 (−2.93, −0.13)	−1.27 (−3.43, 0.8)
IFXAZA	−0.45 (−2.2, 1.2)	0.14 (−1.82, 2.02)	−1.29 (−2.94, 0.3)	−0.99 (−3.27, 1.25)	−0.66 (−2.14, 0.72)	−1.18 (−2.97, 0.56)	−0.51 (−2.27, 1.19)	−0.01 (−2.11, 2.01)	0.49 (−0.88, 1.96)	IFXAZA	0.36 (−1.49, 2.18)	−1.13 (−2.88, 0.49)	0.42 (−1.97, 2.94)	−0.7 (−2.26, 0.75)	−0.93 (−3.07, 1.25)	−0.26 (−2.39, 1.81)	0.15 (−1.99, 2.2)	−0.95 (−2.8, 0.78)	−0.77 (−3.17, 1.61)
MTX	−0.81 (−2.37, 0.68)	−0.22 (−2.02, 1.52)	−1.65 (−3.15, −0.19)	−1.35 (−3.53, 0.78)	−1.02 (−2.41, 0.29)	−1.54 (−3.16, 0.05)	−0.87 (−2.45, 0.67)	−0.37 (−2.4, 1.58)	0.14 (−1.24, 1.58)	−0.36 (−2.18, 1.49)	MTX	−1.5 (−3.06, −0.01)	0.07 (−2.34, 2.54)	−1.06 (−2.42, 0.21)	−1.28 (−3.31, 0.76)	−0.61 (−2.61, 1.31)	−0.21 (−2.21, 1.72)	−1.31 (−2.99, 0.29)	−1.12 (−3.42, 1.14)
NTZ	0.68 (−0.38, 1.78)	1.27 (−0.12, 2.71)	−0.16 (−1.18, 0.92)	0.14 (−1.71, 2.07)	0.47 (−0.59, 1.57)	−0.05 (−1.19, 1.19)	0.62 (−0.48, 1.8)	1.13 (−0.6, 2.88)	1.63 (0.45, 3)	1.13 (−0.49, 2.88)	1.5 (0.01, 3.06)	NTZ	1.56 (−0.7, 4.01)	0.43 (−0.31, 1.2)	0.21 (−1.42, 2)	0.88 (−0.73, 2.52)	1.28 (−0.33, 2.95)	0.18 (−1.03, 1.41)	0.37 (−1.59, 2.41)
NTZIFX	−0.88 (−3.32, 1.39)	−0.29 (−2.88, 2.17)	−1.72 (−4.08, 0.5)	−1.41 (−4.25, 1.3)	−1.08 (−3.43, 1.09)	−1.61 (−4.08, 0.74)	−0.94 (−3.39, 1.38)	−0.43 (−3.19, 2.17)	0.08 (−1.96, 2.03)	−0.42 (−2.94, 1.97)	−0.07 (−2.54, 2.34)	−1.56 (−4.01, 0.7)	NTZIFX	−1.13 (−3.44, 1.02)	−1.35 (−4.08, 1.33)	−0.68 (−3.44, 1.9)	−0.28 (−3.01, 2.3)	−1.38 (−3.9, 0.97)	−1.19 (−4.14, 1.66)
P	0.25 (−0.52, 1.04)	0.85 (−0.34, 2.05)	−0.58 (−1.3, 0.17)	−0.28 (−2, 1.48)	0.05 (−0.72, 0.82)	−0.47 (−1.37, 0.48)	0.19 (−0.63, 1.07)	0.7 (−0.86, 2.28)	1.2 (0.27, 2.3)	0.7 (−0.75, 2.26)	1.06 (−0.21, 2.42)	−0.43 (−1.2, 0.31)	1.13 (−1.02, 3.44)	P	−0.22 (−1.71, 1.38)	0.46 (−1.01, 1.9)	0.85 (−0.59, 2.33)	−0.25 (−1.21, 0.72)	−0.06 (−1.88, 1.82)
PED	0.47 (−1.03, 1.88)	1.07 (−0.85, 2.87)	−0.37 (−2.11, 1.31)	−0.07 (−2.4, 2.23)	0.27 (−1.52, 1.93)	−0.25 (−1.65, 1.06)	0.41 (−1.38, 2.13)	0.92 (−1.33, 3.07)	1.42 (−0.4, 3.28)	0.93 (−1.25, 3.07)	1.28 (−0.76, 3.31)	−0.21 (−2, 1.42)	1.35 (−1.33, 4.08)	0.22 (−1.38, 1.71)	PED	0.67 (−1.47, 2.68)	1.07 (−1.05, 3.08)	−0.03 (−1.91, 1.74)	0.16 (−2.27, 2.53)
SSZ	−0.2 (−1.75, 1.38)	0.39 (−1.05, 1.86)	−1.04 (−2.63, 0.62)	−0.75 (−2.98, 1.56)	−0.41 (−2.04, 1.24)	−0.93 (−2.56, 0.79)	−0.26 (−1.9, 1.45)	0.24 (−1.88, 2.4)	0.74 (−0.94, 2.62)	0.26 (−1.81, 2.39)	0.61 (−1.31, 2.61)	−0.88 (−2.52, 0.73)	0.68 (−1.9, 3.44)	−0.46 (−1.9, 1.01)	−0.67 (−2.68, 1.47)	SSZ	0.4 (−1.13, 1.94)	−0.7 (−2.43, 1.03)	−0.51 (−2.83, 1.86)
SSZ6MP	−0.6 (−2.16, 0.97)	−0.01 (−1.45, 1.45)	−1.44 (−3.05, 0.21)	−1.14 (−3.4, 1.17)	−0.8 (−2.46, 0.84)	−1.33 (−2.98, 0.37)	−0.66 (−2.32, 1.04)	−0.16 (−2.3, 2)	0.35 (−1.36, 2.21)	−0.15 (−2.2, 1.99)	0.21 (−1.72, 2.21)	−1.28 (−2.95, 0.33)	0.28 (−2.3, 3.01)	−0.85 (−2.33, 0.59)	−1.07 (−3.08, 1.05)	−0.4 (−1.94, 1.13)	SSZ6MP	−1.1 (−2.85, 0.63)	−0.91 (−3.25, 1.44)
UST	0.5 (−0.73, 1.74)	1.09 (−0.42, 2.63)	−0.34 (−1.53, 0.89)	−0.04 (−2, 1.98)	0.29 (−0.93, 1.54)	−0.23 (−1.54, 1.13)	0.44 (−0.82, 1.74)	0.95 (−0.88, 2.79)	1.45 (0.13, 2.93)	0.95 (−0.78, 2.8)	1.31 (−0.29, 2.99)	−0.18 (−1.41, 1.03)	1.38 (−0.97, 3.9)	0.25 (−0.72, 1.21)	0.03 (−1.74, 1.91)	0.7 (−1.03, 2.43)	1.1 (−0.63, 2.85)	UST	0.19 (−1.87, 2.29)
VDZ	0.32 (−1.71, 2.3)	0.9 (−1.29, 3.08)	−0.53 (−2.52, 1.45)	−0.22 (−2.75, 2.3)	0.11 (−1.92, 2.09)	−0.41 (−2.48, 1.64)	0.26 (−1.8, 2.28)	0.76 (−1.66, 3.18)	1.27 (−0.8, 3.43)	0.77 (−1.61, 3.17)	1.12 (−1.14, 3.42)	−0.37 (−2.41, 1.59)	1.19 (−1.66, 4.14)	0.06 (−1.82, 1.88)	−0.16 (−2.53, 2.27)	0.51 (−1.86, 2.83)	0.91 (−1.44, 3.25)	−0.19 (−2.29, 1.87)	VDZ

*5ASA, mesalazine; BUD, budesonide; AZA, azathioprine; 6MP, mercaptopurine; MTX, methotrexate; IFX, infliximab; ADA, adalimumab; CZP, certolizumab pegol; NTZ, natalizumab; VDZ, vedolizumab; UST, ustekinumab; SSZ, sulfasalazine; EVE, everolimus; PED, prednisolone; P, placebo*.

The node-splitting analysis of inconsistency for withdrawals was reported in the Supplementary Table 14.

## Discussion

We conducted a systematic review and network meta-analysis of non-biological agents and biological agents for induction and maintenance of remission in adults with CD. Only head-to-head trials was identified in our network meta-analysis, and the node-splitting analysis was used to assess the consistency of the evidence. The purpose of this research is to help making decisions, as well as providing clinicians and patients with medication reference. For patients with prior exposure to anti-TNF agents would have a great impact on the follow-up treatment effect, we divided the treatments into first-line therapy subgroup (with prior exposure to anti-TNF agents) and second-line therapy subgroup (without prior exposure to anti-TNF agents).

From the results in the first-line therapy for induction of remission, we could find that AZA, IFX, IFX+AZA, IFX+MTX were different from placebo for inducing remission. This finding was inconsistent with the results in the previous study (15), where AZA was not different from placebo for induction of remission in Crohn's disease. This might be because there were more clinical trials included in this study which led to different results. Moreover, although first-line therapy IFX+MTX had a higher-ranking probability, it was based on high bias risk studies. The sensitivity analysis which excluded 6 trials with high bias risk found that there was no significant difference between IFX + MTX and placebo. Therefore, only the combination medication (IFX + AZA) performed better than biologic agent monotherapy or non-biologic agent monotherapy. From the results in the second-line therapy for induction of remission, we could find that ADA might be a more efficacious treatment option, which should be interpreted cautiously because the credible interval was wide. Besides, for the time point in defining remission for included induction trials varied, induction studies should be compared carefully. We suggest that future induction trials use the same definition of remission to reduce intrinsic differences in study designs.

Non-biological agents such as AZA have always been common medicines for maintenance of remission in CD, and they also showed superiority in our research, which was consistent with clinical practice. Besides, in this network meta-analysis, ADA were superior to placebo for maintenance of remission, and there was no difference between AZA and ADA. In the sensitivity analysis, when we included those trials whose time points were 1 year or longer, the results were the same as the primary one. Therefore, it could be found that the biological agents were not always better than non-biologic agents for maintenance of remission in CD.

Patients with CD were less likely to have trial withdrawal with ADA, BUD, and NTZ relative to placebo. Besides, we also find this result was consistent with the result when we only included the withdrawals due to the adverse events. In contrast, we observed that the 6-MP and MTX were associated with higher WDAEs compared with placebo. However, these data should be analyzed carefully because randomized controlled trials have insufficient power to detect small but important rare adverse events.

Rational drug use for CD can significantly improve the quality of life of patients and reduce hospitalizations and operations. The appearance of biological agents provides more drugs are available for patients, but it also increases the difficulty of choice. In recent years, various biological agents have been paid more attention. However, according to our research, we have found that non-biological agents also had very good effects in the treatment of CD. Even the combination use of biological agent and non-biological agent was better than biological agent monotherapy. One network meta-regression published previously by Singh et al. (73) compared some biological agents for the induction and maintenance of remission, the results from their paper were consistent with our research.

For VDZ and UST, which have been approved for the treatment of CD recent years, we approved that they had significant efficacy compared with placebo in the induction and maintenance of remission, but due to insufficient sample size, their efficacy should be treated carefully. The highlight was that for patients who previously failed anti-TNF agents, these two drugs provided a new choice for patients because their mechanism was different from that of anti-TNF agents.

There also existed some limits in our research. Due to the lack of head-to-head trials and reliance on only indirect evidence (such as NTZ and ADA) resulted in wide 95% confidence intervals. In addition, in the node-splitting analysis of inconsistency, direct and indirect comparison results of 5ASA-6MP and 6MP-placebo were significantly different (Supplementary Table 8), which showed that for the induction of remission, the result about 6MP was questionable. Another concern with network meta-analysis was heterogeneity between trials, such as the inclusion criteria of patients, risk of bias, the severity of disease. Although we have divided the most important factors into subgroups, we could only perform descriptive analysis in the characteristics table for other heterogeneities. Finally, as our research was based on head-to-head clinical trials, both dosage and compliance were different from the real-world environment to a certain extent. For example, the different strategies of treating CD such as top-down approach and step-up approach could not be compared in our research.

## Conclusion

For induction of remission, azathioprine, infliximab, infliximab + azathioprine were more effective in first-line therapy. In second-line therapy, adalimumab was more effective but should be interpreted carefully. For maintenance of remission adalimumab and azathioprine were more effective. Besides, adalimumab, budesonide, natalizumab had lower withdrawals. Therefore, biological agents were not always better than non-biological agents and they have their own advantages in different treatment methods of Crohn's disease.

## Data Availability Statement

The original contributions presented in the study are included in the article/Supplementary Material, further inquiries can be directed to the corresponding author/s.

## Author Contributions

MR, AM, and HL: conception of the study. MR, ZF, and YW: literature search and data extraction. FS and RM: statistical analysis. MR and YS: drafting the manuscript. AM and HL: revising and completion of final work. All authors reviewed and approved the final manuscript.

## Conflict of Interest

The authors declare that the research was conducted in the absence of any commercial or financial relationships that could be construed as a potential conflict of interest.

## Publisher's Note

All claims expressed in this article are solely those of the authors and do not necessarily represent those of their affiliated organizations, or those of the publisher, the editors and the reviewers. Any product that may be evaluated in this article, or claim that may be made by its manufacturer, is not guaranteed or endorsed by the publisher.
