# Supplementary material for: Will the Inducing and Maintaining Remission of Non-biological Agents and Biological Agents Differ for Crohn's Disease? The Evidence From the Network Meta-Analysis
Source: Front Med (Lausanne). 2021 Sep 1;8:679258. doi: 10.3389/fmed.2021.679258 (PMC8440847; doi:10.3389/fmed.2021.679258)
Supplement: Supplementary file 1 [file Table_1.DOCX]

Provide an explicit statement of questions being addressed, with reference to participants, interventions, comparisons, outcomes, and study design (PICOS).

Participants/population:

We will include adult people (age >= 18, no upper age limit, no restriction in setting, gender, ethnicity) with Crohn’s disease. Studies about pediatric Crohn’s disease was not include because there existed significant difference pediatric and adult CD. According to the study of Judith Kelsen et., the patients less than 5 years of age tend to have significant large bowel involvement. In addition, this group may have more significant diarrhea at presentation (91%) than older children or adults (65%). A major difference between adult and pediatric CD lies in the fact that the impact of inadequate nutrition is more apparent in a growing child or adolescent. This leads to a risk of growth failure, which may precede the onset of intestinal symptoms by years. In addition, delayed puberty can have an important impact on the self-esteem of the adolescent patient and diminish final adult height. This can lead to significant psychosocial complications in the adolescent patient who feels different from their peers. For these differences, the treatments are different between adult and pediatric CD. In our study, patients were divided into first-line treatment and second-line treatment according to whether they had received anti-TNF agents before, because whether patients treated with biological agents had previously received treatment with anti-TNF agents would have an impact on the efficacy of the treatment.

Intervention(s), exposure(s):

We will include almost all commonly used non-biological agents (mesalazine, budesonide, azathioprine, sulfasalazine (SSZ), everolimus (EVE), olsalazine (OLS), mercaptopurine, methotrexate), all biological agents available in Europe or the US (infliximab, adalimumab, certolizumab pegol, vedolizumab, ustekinumab, natalizumab) and placebo.

Non-biological agents: anti-inflammatory agents (such as mesalazine (5ASA), steroids (such as budesonide (BUD), prednisolone (PED)), immunosuppressive agents (such as azathioprine (AZA) and mercaptopurine (6MP), methotrexate (MTX)) are in some countries such as the US nowadays the most frequently prescribed compounds they are overall more costly and have a certain effect. Biologic therapies: anti-tumor necrosis factor (TNF) (such as infliximab (IFX), adalimumab (ADA), certolizumab pegol (CZP)), anti-α4β7 integrin (natalizumab (NTZ)), anti-alpha-4 integrin (vedolizumab (VDZ)) and anti-interleukin 12/23 (ustekinumab (UST)) are also commonly used treatments. Although they are more expensive, they have good effects and less side effects based on previous evidence.

One novel aspect of the network meta-analysis is the inclusion of both non-biological agents and biological agents. We hope to explore the performance of these two categories of drugs in the treatment of CD.

Comparator(s)/control

Where relevant, give details of the alternatives against which the main subject/topic of the review will be compared (e.g. another intervention or a non-exposed control group). In a network meta-analysis each treatment is compared with each other, therefore any treatment can be the comparator.

But placebo is a natural reference that will be used in our occasion.

Primary outcome(s)

Give the most important outcomes.

The primary outcome was remission, which was defined as Crohn’s Disease Activity Index (CDAI) less than 150.

1. Induction of remission

We included trials assessing the induction of remission of non-biological and biological agents between 2 and 18 weeks.

1. Maintenance of remission

We include trials assessing the maintenance of remission of remission with at least 24 weeks in duration.

1. Withdrawals

Secondary end point was total withdrawals which was defined as the total number of patients who were withdrawn from the research after randomization for any reason.

Study design:

Randomized controlled trials
