# Supplementary material for: Will the Inducing and Maintaining Remission of Non-biological Agents and Biological Agents Differ for Crohn's Disease? The Evidence From the Network Meta-Analysis
Source: Front Med (Lausanne). 2021 Sep 1;8:679258. doi: 10.3389/fmed.2021.679258 (PMC8440847; doi:10.3389/fmed.2021.679258)
Supplement: Supplementary file 3 [file Table_3.DOCX]

Supplementary Methods

Search Strategy

**Ovid MEDLINE**

| 1 | (random$.tw. or factorial$.tw. or placebo$).tw. | 1290577 |
| --- | --- | --- |
| 2 | double blind.af. | 200739 |
| 3 | (double$ adj blind$).tw. | 154492 |
| 4 | (assign$.tw. or allocat$).tw. | 446393 |
| 5 | exp randomized controlled trial/ | 520822 |
| 6 | 1 or 2 or 3 or 4 or 5 | 1662589 |
| 7 | (animals not humans).sh. | 4737365 |
| 8 | 6 not 7 | 1576685 |
| 9 | (infliximab or Remicade or monoclonal antibody cA2).mp. | 15471 |
| 10 | (adalimumab or Humira or antibody D2E7).mp. | 8906 |
| 11 | (certolizumab or Cimzia or CDP870).mp. | 1352 |
| 12 | (mercaptopurine or 6-mercaptopurine or 6-thiohypoxanthine or azathioprine or Imuran or Leupurin or Purimethol).mp. | 29370 |
| 13 | (methotrexate or amethopterin or Mexate).mp. | 55373 |
| 14 | (mesalamine or mesalazine or 5-aminosalicylic acid or Asacol or Asacolon or Canasa or Salofalk or Lixacol or Pentasa or Rowasa).mp. | 5318 |
| 15 | (prednisone or delta-cortisone or sterapred or Cortan or Panafcort or Prednidib or Panasol or Orasone or Meticorten).mp. | 54052 |
| 16 | (6-methylprednisolone or Metipred or Urbason or Medrol).mp. | 563 |
| 17 | (sulfasalazine or salicylazosulfapyridine or sulphasalazine or salazosulfapyridine or Azulfadine or Salazopyrin).mp. | 6486 |
| 18 | (natalizumab or Tysabri or Antegren).mp. | 2695 |
| 19 | 9 or 10 or 11 or 12 or 13 or 14 or 15 or 16 or 17 or 18 | 151721 |
| 20 | exp enteritis/ | 13566 |
| 21 | exp Crohn disease/ | 39394 |
| 22 | (inflammatory bowel disease* or crohn*).tw. | 80348 |
| 23 | 20 or 21 or 22 | 97406 |
| 24 | 8 and 19 and 23 | 1374 |

**EMBASE**

| #1 | random$ OR factorial$ OR placebo$:ab,kw,ti | 697488 |
| --- | --- | --- |
| #2 | 'double blind' | 259357 |
| #3 | double$ NEAR/1 blind$ | 259447 |
| #4 | assign$ OR allocat$:ab,kw,ti | 33616 |
| #5 | randomized controlled trial'/exp | 637874 |
| #6 | #1 OR #2 OR #3 OR #4 OR #5 | 1259456 |
| #7 | animals NOT humans | 808650 |
| #8 | #6 NOT #7 | 1239924 |
| #9 | infliximab OR remicade OR 'monoclonal antibody cA2':ab,kw,ti | 53228 |
| #10 | adalimumab OR Humira OR 'antibody D2E7':ab,kw,ti | 35716 |
| #11 | certolizumab OR Cimzia OR CDP870:ab,kw,ti | 7599 |
| #12 | mercaptopurine OR 6-mercaptopurine OR 6-thiohypoxanthine OR azathioprine OR Imuran OR Leupurin OR Purimethol:ab,kw,ti | 118800 |
| #13 | methotrexate OR amethopterin OR Mexate:ab,kw,ti | 189550 |
| #14 | mesalamine OR mesalazine OR '5-aminosalicylic acid' OR Asacol OR Asacolon OR Canasa OR Salofalk OR Lixacol OR Pentasa OR Rowasa:ab,kw,ti | 19364 |
| #15 | prednisone OR delta-cortisone OR sterapred OR Cortan OR Panafcort OR Prednidib OR Panasol OR Orasone OR Meticorten:ab,kw,ti | 187449 |
| #16 | 6-methylprednisolone OR Metipred OR Urbason OR Medrol:ab,kw,ti | 1659 |
| #17 | sulfasalazine OR salicylazosulfapyridine OR sulphasalazine OR salazosulfapyridine OR Azulfadine OR Salazopyrin:ab,kw,ti | 26829 |
| #18 | natalizumab OR Tysabri OR Antegren:ab,kw,ti | 11047 |
| #19 | #9 OR #10 OR #11 OR #12 OR #13 OR #14 OR #15 OR #16 OR #17 OR #18 | 468990 |
| #20 | enteritis'/exp | 308750 |
| #21 | crohn disease'/exp | 96653 |
| #22 | inflammatory bowel disease*' OR crohn*:ab,kw,ti | 146407 |
| #23 | #20 OR #21 OR #22 | 340442 |
| #24 | #8 AND #19 AND #23 | 4173 |

**Cochrane Library**

| #1 | (random$ OR factorial$ OR placebo$):ab,kw,ti | 369113 |
| --- | --- | --- |
| #2 | double blind' | 322199 |
| #3 | double$ near/1 blind$ | 320213 |
| #4 | (assign$ or allocat$):ab,kw,ti | 2132 |
| #5 | randomized controlled trial | 1031610 |
| #6 | #1 or #2 or #3 or #4 or #5 | 1166028 |
| #7 | animals not humans | 2037 |
| #8 | #6 not #7 | 1164443 |
| #9 | (infliximab or Remicade or 'monoclonal antibody cA2'):ti,ab,kw | 2367 |
| #10 | (adalimumab or Humira or 'antibody D2E7'):ti,ab,kw | 3158 |
| #11 | (certolizumab or Cimzia or CDP870):ti,ab,kw | 674 |
| #12 | (mercaptopurine or '6 mercaptopurine' or '6 thiohypoxanthine' or azathioprine or Imuran or Leupurin or Purimethol):ti,ab,kw | 3874 |
| #13 | (methotrexate or amethopterin or Mexate):ti,ab,kw | 11502 |
| #14 | (mesalamine or mesalazine or '5 aminosalicylic acid' or Asacol or Asacolon or Canasa or Salofalk or Lixacol or Pentasa or Rowasa):ti,ab,kw | 1513 |
| #15 | (prednisone or 'delta cortisone' or sterapred or Cortan or Panafcort or Prednidib or Panasol or Orasone or Meticorten):ti,ab,kw | 9690 |
| #16 | ('6 methylprednisolone' or Metipred or Urbason or Medrol):ti,ab,kw | 2287 |
| #17 | (sulfasalazine or salicylazosulfapyridine or sulphasalazine or salazosulfapyridine or Azulfadine or Salazopyrin):ti,ab,kw | 1444 |
| #18 | (natalizumab or Tysabri or Antegren):ti,ab,kw | 412 |
| #19 | #9 or #10 or #11 or #12 or #13 or #14 or #15 or #16 or #17 or #18 | 29397 |
| #20 | enteritis | 965 |
| #21 | crohn disease | 5154 |
| #22 | ('inflammatory bowel disease*' OR crohn*):ti,ab,kw | 6974 |
| #23 | #20 or #21 or #22 | 7794 |
| #24 | #8 and #19 and #23 | 1728 |
