# Supplementary material for: Will the Inducing and Maintaining Remission of Non-biological Agents and Biological Agents Differ for Crohn's Disease? The Evidence From the Network Meta-Analysis
Source: Front Med (Lausanne). 2021 Sep 1;8:679258. doi: 10.3389/fmed.2021.679258 (PMC8440847; doi:10.3389/fmed.2021.679258)
Supplement: Supplementary file 4 [file Table_4.DOCX]

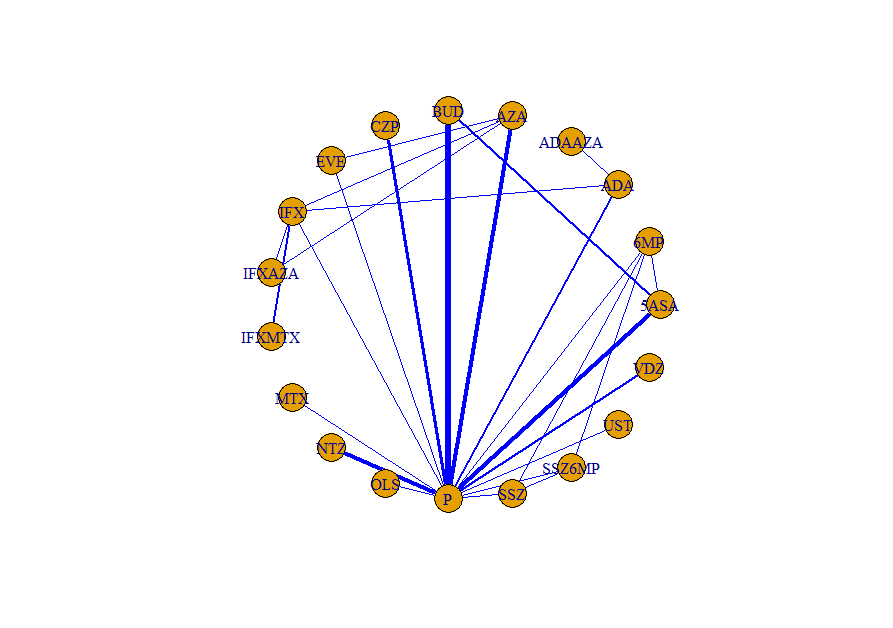


FigS1. Model structure for the induction of remission in first-line therapy


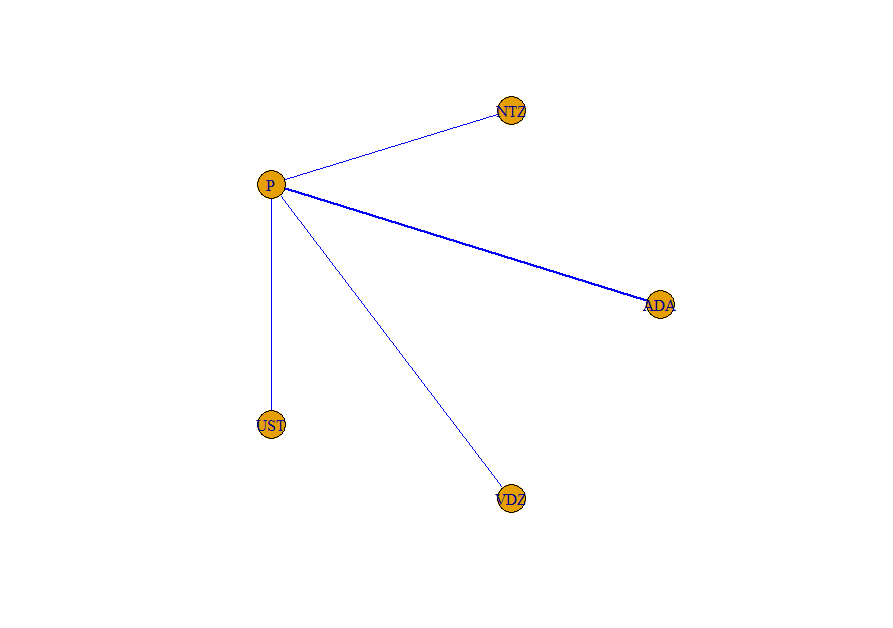


FigS2. Model structure for the induction of remission in second-line therapy


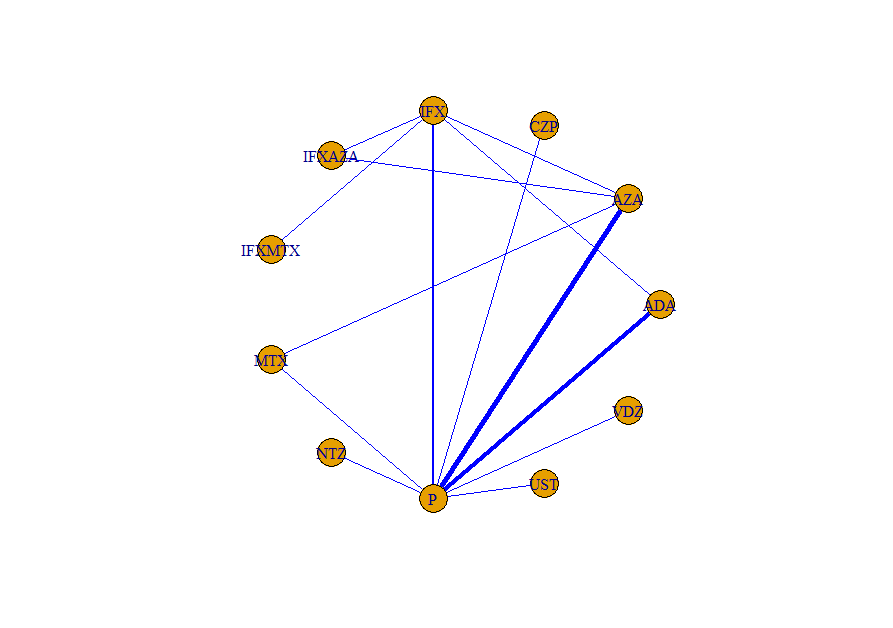


FigS3. Model structure for the maintenance of remission


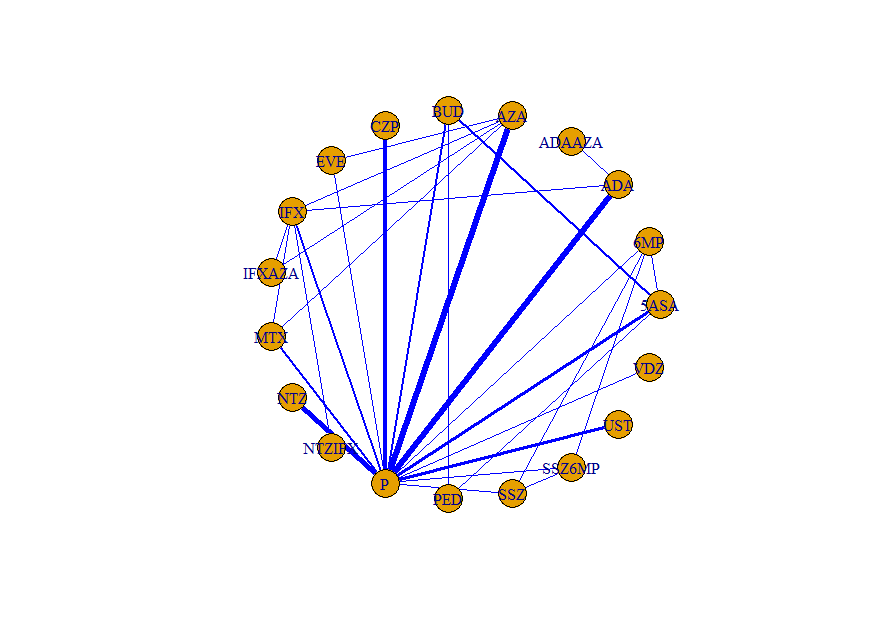


FigS4. Model structure for the withdrawal
