# Supplementary material for: Will the Inducing and Maintaining Remission of Non-biological Agents and Biological Agents Differ for Crohn's Disease? The Evidence From the Network Meta-Analysis
Source: Front Med (Lausanne). 2021 Sep 1;8:679258. doi: 10.3389/fmed.2021.679258 (PMC8440847; doi:10.3389/fmed.2021.679258)
Supplement: Supplementary file 5 [file Table_5.DOCX]

Supplementary Table5: Risk of bias assessment

| Study | Random sequence generation | Allocation concealment | Blinding of participants and personnel | Blinding of outcome assessment | Incomplete outcome data | Selective reporting | Other bias | Overall risk of bias |
| --- | --- | --- | --- | --- | --- | --- | --- | --- |
| HANAUER, 2006 | Low | Low | Low | Low | Low | Low | Low | Low |
| Sandborn, 2007 | Low | Unclear | Low | Low | Unclear | Low | Low | Unclear |
| Watanabe, 2012 | Unclear | Unclear | Low | Low | Unclear | Low | Low | Unclear |
| Sandborn, 2011 | Low | Unclear | Low | Unclear | Low | Low | Low | Unclear |
| Schreiber, 2005 | Low | Unclear | Unclear | Low | Low | Low | Low | Unclear |
| Winter, 2004 | Unclear | Unclear | Low | Unclear | Low | Low | Low | Unclear |
| Schreiber, 2007 | Low | Unclear | Low | Unclear | Low | Low | Low | Unclear |
| Targan, 1997 | Low | Unclear | Unclear | Unclear | Low | Low | Low | Unclear |
| Feagan, 2008 | Unclear | Unclear | Low | Low | Low | Low | Low | Unclear |
| Sands, 2014 | Low | Low | Low | Low | Low | Low | Low | Low |
| Ewe, 1993 | Unclear | Unclear | Low | Unclear | Low | Low | Low | Unclear |
| Feagan, 1995 | Unclear | Unclear | Low | Unclear | Low | Low | Low | Unclear |
| Candy, 1995 | Unclear | Unclear | Low | Unclear | Low | Low | Low | Unclear |
| Reinisch, 2008 | Low | Low | Low | Unclear | Low | Low | Low | Unclear |
| Lemann, 2006 | Low | Unclear | Low | Unclear | Low | Low | Low | Unclear |
| Schroder,  2006 | Unclear | High | Low | Unclear | Low | Low | Low | High |
| Feagan, 2014 | Low | Unclear | Low | Unclear | Low | Low | Low | Unclear |
| Colombel, 2010 | Low | Low | Low | Unclear | Low | Low | Low | Unclear |
| Feagan, 2016 | Low | Unclear | Low | Unclear | Low | Low | Low | Unclear |
| Narula, 2016 | Unclear | Unclear | Unclear | Unclear | Low | Low | Low | Unclear |
| Tremaine, 1994 | High | Unclear | Low | Unclear | Low | Low | Low | High |
| Wright, 1995 | Low | Unclear | Low | Unclear | Unclear | Unclear | Low | Unclear |
| Campieri, 1997 | Unclear | Unclear | Low | Unclear | Low | Low | Low | Unclear |
| Greenberg, 1994 | High | Unclear | Low | Unclear | Low | Low | Low | Unclear |
| MARTIN, 1990 | Unclear | Unclear | Low | Unclear | Low | Low | Low | Unclear |
| PRANTERA, 1999 | Low | Unclear | Low | Unclear | Low | Low | Low | Unclear |
| SINGLETON, 1993 | High | Unclear | Low | Unclear | Unclear | Low | Low | High |
| MALCHOW, 1984 | Unclear | Unclear | Low | Unclear | Low | Low | Low | Unclear |
| Suzuki, 2013 | Low | Unclear | Low | Unclear | Low | Low | Low | Unclear |
| Rutgeerts, 1994 | Unclear | Unclear | Low | Unclear | Unclear | Low | Low | Unclear |
| THOMSEN, 1998 | Low | Low | Low | Unclear | Low | Low | Low | Low |
| BAR–MEIR, 1998 | Unclear | Unclear | Low | Unclear | Low | Low | Low | Unclear |
| Tremaine, 2002 | Low | Unclear | Low | Unclear | Low | Low | Low | Unclear |
| IERSSEL | Unclear | Unclear | Low | Unclear | Unclear | Low | Low | Unclear |
| TROMM, 2011 | Low | Unclear | Low | Unclear | Low | Low | Low | Unclear |
| RASMUSSEN, 1987 | Unclear | Unclear | Low | Unclear | Low | Low | Low | Unclear |
| Matsumoto, 2016 | Low | Unclear | High | Unclear | Low | Low | Low | Unclear |
| Sandborn, 2005 | Unclear | Unclear | Low | Low | Low | Low | Low | Unclear |
| Ghosh, 2003 | High | Unclear | Low | Unclear | Low | Low | Low | High |
| TARGAN, 2007 | High | Unclear | Low | High | Low | Low | Low | High |
| GORDON, 2001 | Unclear | Unclear | Low | Unclear | Low | Low | Low | Unclear |
| Sands, 2007 | Unclear | Unclear | Low | Unclear | Low | Low | Low | Unclear |
| RUTGEERTS, 2012 | Unclear | Unclear | Low | Unclear | Low | Low | Low | Unclear |
| COLOMBEL, 2007 | Unclear | Unclear | Low | Unclear | Low | Low | Low | Unclear |
| Sandborn, 2007 | Unclear | Unclear | Low | Low | Low | Low | Low | Unclear |
| Hanauer, 2002 | Unclear | Unclear | Low | Unclear | Low | Low | Low | Unclear |
| RUTGEERTS, 1999 | Unclear | Unclear | Low | Unclear | Low | Low | Low | Unclear |
| Sandborn, 2013 | Low | Unclear | Low | Low | Unclear | Low | Low | Unclear |
| Ardizzone, 2003 | Low | High | High | Low | Low | Low | Low | High |
| PANÉS, 2013 | High | Unclear | Low | Low | Low | Low | Low | High |
| Rosenberg, 1975 | Unclear | Unclear | Low | Low | Unclear | Unclear | Low | Unclear |
| WILLOUGHBY, 1971 | Unclear | Unclear | Low | Low | Unclear | Unclear | Low | Unclear |
| Feagan, 2000 | High | Unclear | Low | Unclear | Low | Low | Low | Unclear |
| Colombel, 2015 | Unclear | Unclear | Low | Unclear | Low | Low | Low | Unclear |
