# Supplementary material for: Will the Inducing and Maintaining Remission of Non-biological Agents and Biological Agents Differ for Crohn's Disease? The Evidence From the Network Meta-Analysis
Source: Front Med (Lausanne). 2021 Sep 1;8:679258. doi: 10.3389/fmed.2021.679258 (PMC8440847; doi:10.3389/fmed.2021.679258)
Supplement: Supplementary file 6 [file Table_6.DOCX]

Supplementary Table 6 Rank probability for induction of remission (First-line therapy)

| Treatment | Probability of ranking first |
| --- | --- |
| 5ASA | 0.0009250 |
| 6MP | 0.0067875 |
| ADA | 0.0022625 |
| ADAAZA | 0.0085000 |
| AZA | 0.0157625 |
| BUD | 0.0003000 |
| CZP | 0.0131250 |
| EVE | 0.0049125 |
| IFX | 0.0182500 |
| IFXAZA | 0.3078375 |
| IFXMTX | 0.3933125 |
| MTX | 0.0854875 |
| NTZ | 0.0036375 |
| OLS | 0.0000625 |
| P | 0.0000000 |
| SSZ | 0.0013250 |
| SSZ6MP | 0.0807250 |
| UST | 0.0390125 |
| VDZ | 0.0177750 |

5ASA, mesalazine; BUD, budesonide; AZA, azathioprine; 6MP, mercaptopurine; MTX, methotrexate; IFX, infliximab; ADA, adalimumab; CZP, certolizumab pegol; NTZ, natalizumab; VDZ, vedolizumab; UST, ustekinumab; SSZ, sulfasalazine; EVE, everolimus; OLS, olsalazine; P, Placebo
