# Supplementary material for: Will the Inducing and Maintaining Remission of Non-biological Agents and Biological Agents Differ for Crohn's Disease? The Evidence From the Network Meta-Analysis
Source: Front Med (Lausanne). 2021 Sep 1;8:679258. doi: 10.3389/fmed.2021.679258 (PMC8440847; doi:10.3389/fmed.2021.679258)
Supplement: Supplementary file 8 [file Table_8.DOCX]

Supplementary Table 8 Node-splitting analysis of inconsistency for induction of remission

|  | t1 | t2 | p |
| --- | --- | --- | --- |
| t1 | 5ASA | 6MP | 0.0036 |
| t11 | 5ASA | BUD | 0.0599 |
| t12 | 5ASA | P | 0.8709 |
| t13 | 6MP | P | 0.0024 |
| t14 | ADA | IFX | 0.3315 |
| t15 | ADA | P | 0.3203 |
| t16 | AZA | IFX | 0.044 |
| t17 | AZA | P | 0.0406 |
| t18 | BUD | P | 0.0598 |
| t19 | IFX | P | 0.2163 |

Cl, confidence interval; 5ASA, mesalazine; BUD, budesonide; AZA, azathioprine; 6MP, mercaptopurine; IFX, infliximab; ADA, adalimumab; P, Placebo
