# Supplementary material for: Will the Inducing and Maintaining Remission of Non-biological Agents and Biological Agents Differ for Crohn's Disease? The Evidence From the Network Meta-Analysis
Source: Front Med (Lausanne). 2021 Sep 1;8:679258. doi: 10.3389/fmed.2021.679258 (PMC8440847; doi:10.3389/fmed.2021.679258)
Supplement: Supplementary file 9 [file Table_9.DOCX]

Supplementary table 9 Rank probability for induction of remission (Second-line therapy)

| Treatment | Probability of ranking first |
| --- | --- |
| ADA | 0.6013750 |
| NTZ | 0.1104500 |
| P | 0.0008875 |
| UST | 0.2145125 |
| VDZ | 0.0727750 |

ADA, adalimumab; NTZ, natalizumab; VDZ, vedolizumab; UST, ustekinumab; P, Placebo
