# Supplementary material for: Will the Inducing and Maintaining Remission of Non-biological Agents and Biological Agents Differ for Crohn's Disease? The Evidence From the Network Meta-Analysis
Source: Front Med (Lausanne). 2021 Sep 1;8:679258. doi: 10.3389/fmed.2021.679258 (PMC8440847; doi:10.3389/fmed.2021.679258)
Supplement: Supplementary file 10 [file Table_10.DOCX]

Supplementary table 10 Rank probability for maintenance of remission

| Treatment | Probability of ranking first |
| --- | --- |
| ADA | 0.0685500 |
| AZA | 0.0227250 |
| CZP | 0.0888625 |
| IFX | 0.0022375 |
| IFXAZA | 0.2592375 |
| IFXMTX | 0.0773125 |
| MTX | 0.0512875 |
| NTZ | 0.2826875 |
| P | 0.0000000 |
| UST | 0.0519250 |
| VDZ | 0.0951750 |

AZA, azathioprine; MTX, methotrexate; IFX, infliximab; ADA, adalimumab; CZP, certolizumab pegol; NTZ, natalizumab; VDZ, vedolizumab; UST, ustekinumab; P, Placebo
