# Supplementary material for: Will the Inducing and Maintaining Remission of Non-biological Agents and Biological Agents Differ for Crohn's Disease? The Evidence From the Network Meta-Analysis
Source: Front Med (Lausanne). 2021 Sep 1;8:679258. doi: 10.3389/fmed.2021.679258 (PMC8440847; doi:10.3389/fmed.2021.679258)
Supplement: Supplementary file 11 [file Table_11.DOCX]

Supplementary Table 11 Node-splitting analysis of inconsistency for maintenance of remission

|  | t1 | t2 | p |
| --- | --- | --- | --- |
| t1 | ADA | IFX | 0.6263 |
| t11 | ADA | P | 0.6299 |
| t12 | AZA | IFX | 0.9183 |
| t13 | AZA | MTX | 0.8961 |
| t14 | AZA | P | 0.9975 |
| t15 | IFX | P | 0.6396 |
| t16 | MTX | P | 0.9142 |

Cl, confidence interval; AZA, azathioprine; MTX, methotrexate; IFX, infliximab; ADA, adalimumab; P, Placebo
