# Supplementary material for: Will the Inducing and Maintaining Remission of Non-biological Agents and Biological Agents Differ for Crohn's Disease? The Evidence From the Network Meta-Analysis
Source: Front Med (Lausanne). 2021 Sep 1;8:679258. doi: 10.3389/fmed.2021.679258 (PMC8440847; doi:10.3389/fmed.2021.679258)
Supplement: Supplementary file 12 [file Table_12.DOCX]

Table S12 Network meta-analysis results for maintenance of remission- sensitivity analyses

AZA, azathioprine; MTX, methotrexate; IFX, infliximab; ADA, adalimumab; CZP, certolizumab pegol; NTZ, natalizumab; VDZ, vedolizumab; UST, ustekinumab; P, Placebo
