# Supplementary material for: Will the Inducing and Maintaining Remission of Non-biological Agents and Biological Agents Differ for Crohn's Disease? The Evidence From the Network Meta-Analysis
Source: Front Med (Lausanne). 2021 Sep 1;8:679258. doi: 10.3389/fmed.2021.679258 (PMC8440847; doi:10.3389/fmed.2021.679258)
Supplement: Supplementary file 14 [file Table_14.DOCX]

Supplementary Table S14 Node-splitting analysis of inconsistency for withdrawals

|  | t1 | t2 | p |
| --- | --- | --- | --- |
| t1 | 5ASA | 6MP | 0.8314 |
| t11 | 5ASA | BUD | 0.5536 |
| t12 | 5ASA | P | 0.9481 |
| t13 | 5ASA | PED | 0.4947 |
| t14 | 6MP | P | 0.8394 |
| t15 | ADA | IFX | 0.3107 |
| t16 | ADA | P | 0.3103 |
| t17 | AZA | IFX | 0.178 |
| t18 | AZA | MTX | 0.2839 |
| t19 | AZA | P | 0.0445 |
| t110 | BUD | P | 0.8305 |
| t111 | BUD | PED | 0.479 |
| t112 | IFX | MTX | 0.4341 |
| t113 | IFX | P | 0.0459 |
| t114 | MTX | P | 0.0647 |

Cl, confidence interval; 5ASA, mesalazine; BUD, budesonide; AZA, azathioprine; 6MP, mercaptopurine; MTX, methotrexate; IFX, infliximab; ADA, adalimumab; NTZ, natalizumab; PED, prednisolone; P, Placebo
