# Supplementary material for: Will the Inducing and Maintaining Remission of Non-biological Agents and Biological Agents Differ for Crohn's Disease? The Evidence From the Network Meta-Analysis
Source: Front Med (Lausanne). 2021 Sep 1;8:679258. doi: 10.3389/fmed.2021.679258 (PMC8440847; doi:10.3389/fmed.2021.679258)
Supplement: Supplementary file 15 [file Data_Sheet_1.PDF]

Study	Trial type	Location	Intervention group	Control group	Number in the Intervention group	Number in the Control group	Definition of remission	Time of primary outcome measurement	Baseline disease severity at the time of randomization- Intervention group	Baseline disease severity at the time of randomization-Control group	Prior exposure to anti-TNF	Concomitant therapy
HANAUER, 2006	Induction	North America<br>Europe	Adalimumab 40 mg/20 mg<br>Adalimumab 40 mg/80 mg<br>Adalimumab 80 mg/160 mg	Placebo	74<br>75<br>76	74	CDAI < 150<br>CDAI < 150<br>CDAI < 150	4	299<br>301<br>295	296	No	Yes(Systemic corticosteroid 20%, Budesonide 12.7%, Azathioprine 15.4%, 6-Mercaptopurine 11.4%, Methotrexate 3.0%, Crohn's disease–related antibiotics 8.7%, 5-Aminosalicylates 51.2%)
Sandborn, 2007	Induction	North America<br>Europe	Adalimumab 160/80 mg	Placebo	159	166	CDAI <= 150	4	313	313	Yes(Infliximab 100%)	Yes(Steroids 39.4%, Any immunosuppressive agent 48.6%, 5-aminosalicylates 32.3%)
Watanabe, 2012	Induction	Japan	Adalimumab 160/80 mg<br>Adalimumab 80/40 mg	Placebo	33<br>34	23	CDAI < 150	4	300.5<br>302.7	308.1		Yes(Aminosalicylates 91.1%, Immunosuppressants 32.2%, Corticosteroids 21.1% 5.6%, Enteral nutrition 67.8%, Anti-TNF57.8%)
	Maintenance	Japan	Adalimumab 40 mg	Placebo	25	25	CDAI < 150	52	325.5	296.7		Yes(Aminosalicylates 88%, Immunosuppressants 36%, Corticosteroids 16%, CD-related antibiotics 4%, Enteral nutrition 58%, Anti-TNF 54%)
Sandborn, 2011	Induction	Multinational	Certolizumab 400 mg	Placebo	215	209	CDAI <= 150	6	292.7	262.1		Yes(Corticosteroid 44.5%, Immunosuppressant 32.9%, Corticosteroid or immunosuppressant 62.3%, Corticosteroid and immunosuppressant 15.1%, Neither corticosteroid nor immunosuppressant 37.7%)
Schreiber, 2005	Induction	North America<br>Europe<br>South Africa	Certolizumab 100mg<br>Certolizumab 200 mg<br>Certolizumab 400 mg	Placebo	74<br>72<br>72	73	CDAI < 150	2, 4, 6, 8, 10, 12	N/A	N/A		Yes(Aminosalicylates 43.3%, Antidiarrheals 19.6%, Anti-infectives 8.9%, Codeine and derivatives 6.2%, Immunomodulators 37.1%, Glucocorticoids 35.7%)
Winter, 2004	Induction	Israel<br>Europe<br>South Africa	Certolizumab 1.25 mg/kg<br>Certolizumab 5 mg/kg<br>Certolizumab 10 mg/kg<br>Certolizumab 20 mg/kg	Placebo	2<br>25<br>17<br>23	25	CDAI <= 150	2	About 310<br>About 310<br>About 310<br>About 310	About 310		Yes(aminosalicylate 43.5%, steroid 28.3%, immunosuppressive 44.6%)
Schreiber, 2007	Maintenance	Multinational	Certolizumab 400 mg	Placebo	215	210	CDAI score <= 151 poin	26	306	301	Yes(24.2%received infliximab)	Yes(Glucocorticoids only 21.4%, Immunosuppressive agents only 26.1%, Glucocorticoids plus immunosuppressive agents 14.6%)
Targan, 1997	Induction	North America<br>Europe	Infliximab 5 mg/kg<br>Infliximab 10 mg/kg<br>Infliximab 20 mg/kg	Placebo	27<br>28<br>28	25	CDAI < 150	12	312<br>318<br>307	288	No	Yes(Prednisone 59.2%, Mercaptopurine 14.8%, Azathioprine 22.2%, Oral aminosalicylates 59.3%)
Feagan, 2008	Induction	Canada	Vedolizumab 2 mg/kg<br>Vedolizumab 0.5 mg/kg	Placebo	62<br>58	58	CDAI < 150	8	296.6<br>288.1	288	No	N/A
Sands, 2014	Induction	Multinational	Vedolizumab 300 mg	Placebo	51<br>158<br>209	50<br>157<br>207	CDAI <= 150	6<br>6<br>6	307.3<br>316.1<br>313.9	286.1<br>306.1<br>301.3	Yes(anti-TNF 100%)<br>Yes(anti-TNF 100%)<br>Yes(anti-TNF 75.7%)	Yes(Corticosteroid 46.5%, Immunosuppressive use 54.5% , Mesalamine use 62.4%)<br>Yes(Corticosteroid 54.3%, Immunosuppressive use 27.0% , Mesalamine use 21.0%)<br>Yes(Corticosteroid 52.4%, Immunosuppressive use 33.7% , Mesalamine use 31%)
Ewe, 1993	Induction	Germany	Azathioprine 2.5 mg/kg/day	Placebo	21	21	CDAI < 150	16	290	285	N/A	Yes(All patients who took steroids before the trial were also undergoing concomitant salazosulfapyridine or mesalazine treatment)
Feagan, 1995	Induction	North America	Methotrexate 25 mg/wk	Placebo	94	47	CDAI <=150	16	181	190	N/A	Yes(Prednisone 100%)
Candy, 1995	Induction	South Africa	Azathioprine 2.5 mg/kg/day	Placebo	33	30	CDAI < 150	12	301	282	N/A	Yes(Prednisone 100%)
	Maintenance	South Africa	Azathioprine 2.5 mg/kg/day	Placebo	33	30	CDAI < 175	60	N/A	N/A	N/A	No
Reinisch, 2008	Induction	Multinational	Azathioprine 2.5 mg/kg/day<br>Everolimus 6 mg/day	Placebo	50<br>60	28	CDAI <150	4	282<br>304	280	No	Yes(Prednisone 100%)
	Maintenance	Multinational	Azathioprine 2.5 mg/kg/day<br>Everolimus 6 mg/day	Placebo	36<br>38	22	CDAI < 150	28	283<br>310	284	No	No
Lemann, 2006	Induction	France	Infliximab 5 mg/kg + Azathioprine or 6-Mercapto	Azathioprine or 6-Mercaptopurine + placebo	57	56	CDAI <=150	24	240	181	No	N/A
Schroder, 2006	Induction	Germany	Infliximab 5 mg/kg + methotrexate 20 mg/wk	Infliximab 5 mg/kg	11	8	CDAI <=150	2, 24, 48	251	293	No	Yes(Corticosteroids 78.9%, 5-Aminosalicylates 26.3%)
Feagan, 2014	Induction	Canada	Infliximab 5 mg/kg + placebo	Infliximab 5 mg/kg + MTX 25 mg	63	63	CDAI < 150	14	207.6	207.8	No	Yes(oral folic acid 100%, prednisone 100%)
Colombel, 2010	Induction	North America<br>Europe<br>Israel	Infliximab 5 mg/kg<br>Azathioprine 2.5 mg/kg/day	infliximab + azathioprine	169<br>170	169	CDAI <150	6, 10, 18	284.8<br>287.2	289.9	No	Yes(Budesonide 14.2%, 5-Aminosalicylic compounds 54.3%)
	Maintenance	North America<br>Europe<br>Israel	Infliximab 5 mg/kg<br>Azathioprine 2.5 mg/kg/day	infliximab + azathioprine	169<br>170	169	CDAI < 150	26, 34, 42, 50	284.8<br>287.2	289.9	No	Yes(Budesonide 14.2%, 5-Aminosalicylic compounds 54.3%)
Feagan, 2016	Induction	Multinational	Ustekinumab 130mg<br>Ustekinumab 6 mg/kg	Placebo	245<br>249	247	CDAI <150	3, 6, 8	321<br>327.6	319	Yes(anti-TNF 100%)	Yes(Immunosuppressant 31.4%, Aminosalicylate 20.8%, Glucocorticoid 45.9%)
	Induction	Multinational	Ustekinumab 130mg<br>Ustekinumab 6 mg/kg	Placebo	209<br>209	210	CDAI < 150	3, 6, 8	304.1<br>302.2	302.2	Yes(anti-TNF 32%)	Yes(Immunosuppressant 34.9%, Aminosalicylate 43.2%, Glucocorticoid 39.3%)
Narula, 2016	Induction	Austria	Infliximab 5 mg/kg	Adalimumab 160 mg	251	111	HBI <5	12	HLB：7	HLB：8	no	yes(5-Aminosalicylates 79.3%, Steroids 29.8%, AZA/MP 40.1%, MTX 2.8%)
	Maintenance	Austria	Infliximab 5 mg/kg	Adalimumab 160 mg	154	82	HBI < 5	52	HLB：7	HLB：8	No	Yes(5-Aminosalicylates 79.3%, Steroids 29.8%, AZA/MP 40.1%, MTX 2.8%)
Tremaine, 1994	Induction	US	Asacol 3.2g/day	Placebo	20	18	CDAI < 150 and at least	16	N/A	N/A	No	Yes(oral steriods 57.9%, steriod enemas 7.9%, sulfasalazine 34.2%, 5-ASA enemas 5.3%, metronidazole 23.7%, azathioprie or 6-MP 0)
Wright, 1995	Induction	Multinational	Olsalazine, 1 g b.i.d.	Placebo	46	45	N/A	16	N/A	N/A	N/A	N/A
Campieri, 1997	Induction	Multinational	Budesonide 9 mg once daily<br>Budesonide 4.5 mg twice daily	Prednisolone 40mg once daliy	58<br>61	58	CDAI <= 150	2, 8	277<br>274	279	N/A	N/A
Greenberg, 1994	Induction	Multinational	BUDESONIDE 3 mg daliy<br>BUDESONIDE 9 mg daliy<br>BUDESONIDE 15 mg daliy	Placebo	67<br>61<br>64	66	CDAI <= 150	8	293<br>296<br>285	287	N/A	N/A
MARTIN, 1990	Induction	Multicenter	5-Aminosalicylic Acid 3 g/day	Standard oral prednisone	19	26	CDAI < 150	12	295	291	N/A	Yes(Concomitant low dosage prednlsone 26%)
PRANTERA, 1999	Induction	Italy	Mesalamine tablets 4 g<br>Mesalamine 4 g	6-Methylprednisolone 40 mg	35<br>28	31	CDAI <= 150	3, 6, 9, 12	220<br>222	233	N/A	N/A
SINGLETON, 1993	Induction	Multinational	Mesalamine at 1 g/day<br>Mesalamine at 2 g/day<br>Mesalamine at 4 g/day	Placebo	80<br>75<br>75	80	CDAI < 151.	16	271<br>265<br>260	277	N/A	N/A
MALCHOW, 1984	Induction	Europe	Sulfasalazine 3 g<br>6-methyl-prednisolone<br>6-methylprednisolone + sulfasalazine 3 g	Placebo	54<br>47<br>56	58	N/A	18	182<br>147<br>185	178	N/A	N/A
Suzuki, 2013	Induction	Japan	Budesonide 9 mg od<br>Budesonide 15 mg od	Placebo	26<br>25	26	CDAI<=150	2, 4, 8	CDAI<300 76.6%; CDAI>=300 23.4%		N/A	Yes(Nutritional therapy 5.2%, 5-ASA 19.5%, Nutritional therapy and 5-ASA 70.1%)
Rutgeerts, 1994	Induction	Europe	Budesonide 9 mg per day	Prednisolone 40 mg per day	88	88	CDAI <= 150	2, 4, 8, 10	275	279	N/A	No
THOMSEN, 1998	Induction	Multinational	Budesonide 9 mg once daily	Mesalamine 2 g twice daily	93	89	CDAI <= 150	8, 12, 16	266	278	N/A	N/A
BAR–MEIR, 1998	Induction	Israel	Budesonide 9 mg once daily	Prednisolone 40 mg once daily	100	101	CDAI < 150	8	264	265	N/A	N/A
Tremaine, 2002	Induction	US	Bbudesonide CIR 9 mg once daily<br>Budesonide CIR 9 mg bid	Placebo	80<br>79	41	CDAI <= 150	2, 4, 8	280<br>279	271	N/A	N/A
IERSSEL	Induction	N/A	Oral budesonide 9 mg/day	Prednisolone 40 mg/day	9	9	CDAI <= 150	10	N/A	N/A	N/A	N/A
TROMM, 2011	Induction	Multinational	Budesonide 3 * 3 mg/day<br>Budesonide 1 * 9 mg/day	Mesalamine 3 * 1.5 g/day	78<br>76	153	CDAI <= 150	8	265.6<br>265.6	267.2	N/A	Yes(AZA/6-MP 3.3%)
RASMUSSEN, 1987	Induction	Denmark	5-Aminosalicylic Acid 1500 mg/day	Placebo	23	27	N/A	16	153	177	N/A	N/A
Matsumoto, 2016	Induction	Japan	Adalimumab	Adalimumab + Azathioprine	85	91	CDAI <= 150	4, 26	276	265	No	Yes(Elemental diet 50%, 5-ASA 69.9%, Steroid use 10.2%)
Sandborn, 2005	Induction	Multinational	Natalizumab 300 mg	Placebo	724	181	CDAI < 150	2, 4, 6, 8, 10, 12	302	303	Yes(anti-TNF 39.8%)	Yes(Corticosteroids 37.7%, Immunosuppressants 33.0%, Antibiotics 6.1%, 5-Aminosalicylates 46.7%, ≥1 Corticosteroids or immunosuppressants 56.0%, Corticosteroids and immunosuppressants 14.7%)
	Maintenance	Multinational	Natalizumab 300 mg	Placebo	130	120	CDAI < 150	36, 60	105	118	Yes(anti-TNF 36.3%)	Yes(Corticosteroids 41.3%, Immunosuppressants 36.0%, Antibiotics 7.4%, 5-Aminosalicylates 49.9%, ≥1 Corticosteroids or immunosuppressants 58.7%, Corticosteroids and immunosuppressants 18.6%)
Ghosh, 2003	Induction	Multinational	Natalizumab 3 mg/kg, one infusion<br>Natalizumab 3 mg/kg, two infusion<br>Natalizumab 6 mg/kg, two infusion	Placebo	68<br>66<br>51	63	CDAI < 150	2, 4, 6, 8, 12	288<br>300<br>298	300	No	Yes(5-Aminosalicylate compounds 57.3%, Oral corticosteroids 52.8%, Azathioprine or mercaptopurine with or without corticosteroids 29.8%)
TARGAN, 2007	Induction	Multinational	Natalizumab 300 mg	Placebo	259	250	CDAI < 150	4, 8, 12	303.9	299.5	Yes(anti-TNF 47.5%)	Yes(5-ASA compounds 48.7%, Immunosuppressants 37.9%, Corticosteroids 39.9%, Antibiotics 5.9%, Diet 1.4%)
GORDON, 2001	Induction	Multinational	Natalizumab	Placebo	18	12	CDAI < 150	2	258	273	N/A	Yes(Prednisolone/budesonide 63.3%, Azathioprine 26.7%, Mesalamine 73.3%, Mesalamine 16.7%)
Sands, 2007	Induction	United States	Natalizumab 300 mg + infliximab	Placebo + Infliximab	52	27	CDAI < 150	2, 4, 6, 8, 10	263.8	243.6	Yes(infliximab 100%)	Yes(5-Aminosalicylate compounds 43.0%, Azathioprine, mercaptopurine, or methotrexate 51.9%, Oral steroids 27.8%, Antibiotics 19.0%)
RUTGEERTS, 2012	Maintenance	North America	Adalimumab 40 mg	Placebo	64	65	CDAI < 150	12, 52	318.7	321.1	Yes(anti-TNF 51.9%)	Yes(Mesalamine 21.7%, Immunomodulators 41.1%, Corticosteroids 26.4%, CD-related antibiotics 10.9%)
COLOMBEL, 2007	Maintenance	North America<br>Europe<br>South Africa<br>Australia	Adalimumab 40 mg<br>Adalimumab 40 mg/wk	Placebo	172<br>157	170	CDAI < 150	26, 56	316.6<br>316.6	316.6	Yes(anti-TNF 50%)	Yes(corticosteroid 42.1%, immunosuppressive agent 48.1%)
Sandborn, 2007	Maintenance	North America<br>Europe	Adalimumab 40 mg<br>Adalimumab 40 mg/wk	Placebo	19<br>18	18	CDAI < 150	56	106<br>88	107	No	Yes(corticosteroid 49.1%, immunosuppressive agent 21.8%, 5-Aminosalicylates 61.8%)
Hanauer, 2002	Maintenance	North America<br>Europe<br>Israel	Infliximab 5 mg/kg<br>Infliximab 10 mg/kg	Placebo	110<br>113	112	CDAI < 150	30	299<br>299	299	No	Yes(5-aminosalicylates 47%, 6-mercaptopurine and azathioprine 24%, Methotrexate 3%)
RUTGEERTS, 1999	Maintenance	North America<br>Europe	Infliximab 10 mg/kg	Placebo	37	36	CDAI < 150	44	220-400	220-400	No	Yes(azathioprine, 6-mercaptopurine, sulfasalazine, or mesalamine)
Sandborn, 2013	Maintenance	Multinational	Vedolizumab 300 mg<br>Vedolizumab 300 mg	Placebo	154<br>154	153	CDAI ≤150	52	N/A	N/A	N/A	N/A
Ardizzone, 2003	Maintenance	Italy	Azathioprine 2 mg/kg/day	Methotrexate 25 mg/wk	27	27	CDAI < 150	12, 24	225.96	213.36	N/A	Yes(prednisolone 100%)
PANÉS, 2013	Maintenance	Spain	Azathioprine 2.5 mg/kg/day	Placebo	68	63	CDAI <= 150	76	91.3	114.5	No	Yes(corticosteroids 69.5%)
Rosenberg, 1975	Maintenance	United States	Azathioprine 2 mg/kg	Placebo	10	10	N/A	26	N/A	N/A	N/A	N/A
WILLOUGHBY, 197	Maintenance	United Kingdor	Azathioprine 2 mg/kg/day	Placebo	11	11	N/A	24	N/A	N/A	N/A	Yes(prednisolone 100%, prochlorperazine 100%)
Feagan, 2000	Maintenance	Canada<br>United States	Intramuscular MTX 15 mg/wk	Placebo	40	36	CDAI <= 150	40	94	84	N/A	Yes(prednisolone 42.1%)
Colombel, 2015	Maintenance	Multinational	Infliximab monotherapy<br>Azathioprine monotherapy	infliximab 5 mg/kg +azathioprine 2.5mg/kg	62<br>54	72	CDAI < 150	26	286.8<br>286.8	286.8	No	Yes(Systemic corticosteroids 100%, Budesonide 11.2%, 5-ASA compounds 56.4%)
