# Supplementary material for: Will the Inducing and Maintaining Remission of Non-biological Agents and Biological Agents Differ for Crohn's Disease? The Evidence From the Network Meta-Analysis
Source: Front Med (Lausanne). 2021 Sep 1;8:679258. doi: 10.3389/fmed.2021.679258 (PMC8440847; doi:10.3389/fmed.2021.679258)
Supplement: Supplementary file 16 [file Data_Sheet_2.PDF]

Figures

Figure 3

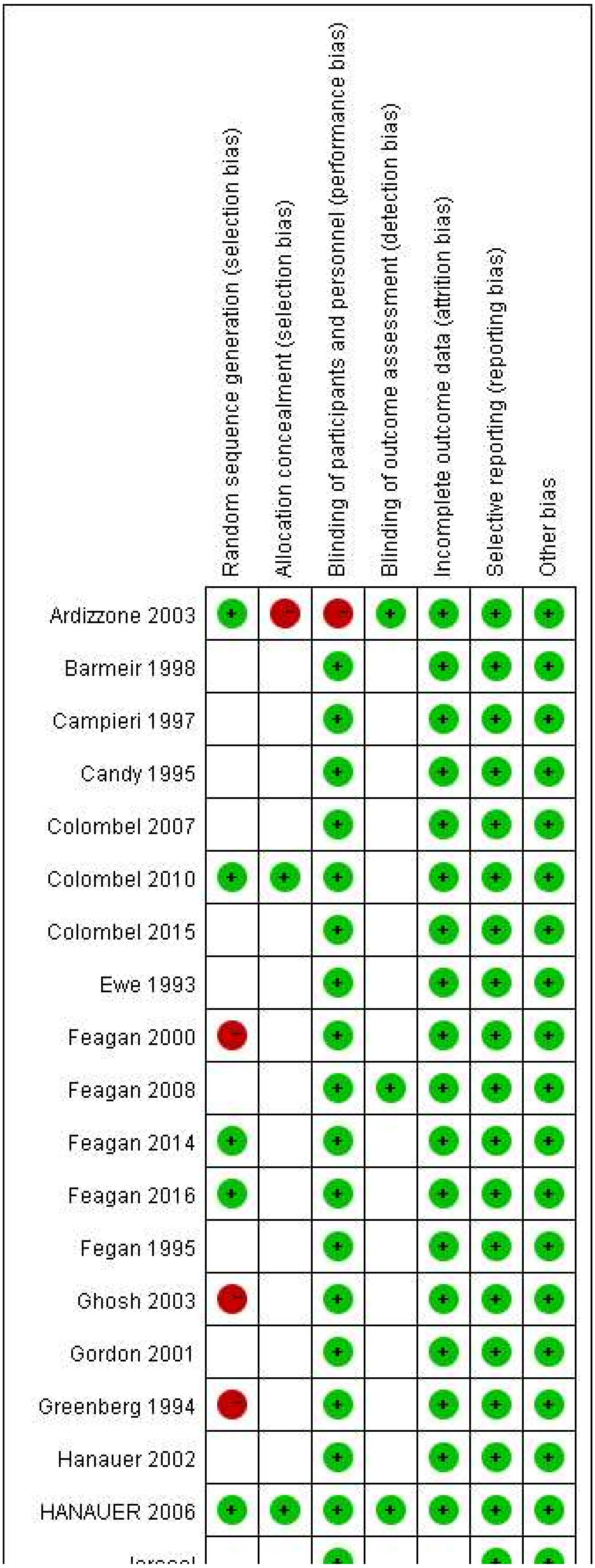

[illegible]			+			+	+
Lemann 2006	+		+		+	+	+
Malchow 1984			+		+	+	+
Martin 1990			+		+	+	+
Matsumoto 2016	+		−		+	+	+
Narula 2016					+	+	+
Panes 2013	−		+	+	+	+	+
Pranrera 1999	+		+		+	+	+
Rasmussen 1987			+		+	+	+
Reinisch 2008	+	+	+		+	+	+
Rosenberg 1975			+	+			+
Rutgeerts 1994			+			+	+
Rutgeerts 1999			+		+	+	+
Rutgeerts 2012			+		+	+	+
Sandborn 2005			+	+	+	+	+
Sandborn 2007	+		+	+	+	+	+
Sandborn 2011	+		+		+	+	+
Sandborn 2013	+		+	+		+	+
Sands 2007			+		+	+	+
Sands 2014	+	+	+	+	+	+	+
Schreiber 2005	+			+	+	+	+
Schreiber 2007	+		+		+	+	+
Schroder 2006		−	+		+	+	+
Singleton 1993	−		+			+	+
Suzuki 2013	+		+		+	+	+
Targan 1997	+					+	+
Targan 2007	−		+	−	+	+	+
Thomsen 1998	+	+	+		+	+	+
Tremaine 1994	−		+		+	+	+
Tremaine 2002	+		+		+	+	+
Tromm 2011	+		+		+	+	+
Watanabe 2012			+	+		+	+
Willoughby 1971			+	+		+	+

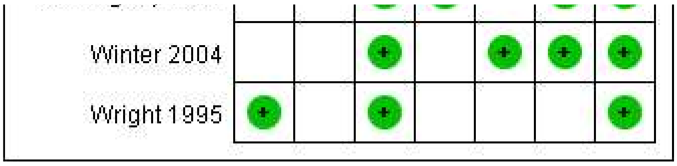

Risk of bias summary: review authors' judgements about each risk of bias item for each included study.
